# Differential protein expression in diverse brain areas of Parkinson’s and Alzheimer’s disease patients

**DOI:** 10.1038/s41598-020-70174-z

**Published:** 2020-08-04

**Authors:** A. R. Esteves, S. M. Cardoso

**Affiliations:** 1 CNC–Center for Neuroscience and Cell Biology, University of Coimbra, Largo Marquês de Pombal, 3004-517 Coimbra, Portugal; 2 CIBB-Centre for Innovative Biomedicine and Biotechnology, University of Coimbra, Coimbra, Portugal; 3 Institute of Cellular and Molecular Biology, Faculty of Medicine, University of Coimbra, Coimbra, Portugal

**Keywords:** Neurological disorders, Autophagy, Cytoskeleton, Protein folding

## Abstract

Many hypotheses have been postulated to define the etiology of sporadic Parkinson’s and Alzheimer’s disorders (PD and AD) but there is no consensus on what causes these devastating age-related diseases. Braak staging of both pathologies helped researchers to better understand the progression and to identify their prodromal and symptomatic phases. Indeed, it is well accepted that Lewy body pathology and neurofibrillary tangles appearance correlates with disease progression and severity of symptoms in PD and AD, respectively. Additionally, several studies in PD and AD models try to disclose which cellular mechanisms are defaulted and trigger the neurodegenerative process that culminates with neuronal death causing PD and AD classical symptomatology. Herein, we determined expression levels of proteins involved in microtubule assembly, autophagic-lysosomal pathway and unfolded protein response in the cortex, hippocampus and SNpc of PD and AD patients, vascular dementia patients and aged-match controls. The differential expression allowed us to determine which pathways are determinant to synaptic dysfunction and to establish a time line for disease progression. Our results allow us to challenge the hypothesis that both PD and AD pathologies are caused by α-synuclein or Aβ pathology propagation throughout the brain in a prion-like manner.

## Introduction

Aged-related neurodegenerative disorders with unknown etiology, such as sporadic Alzheimer’s (AD) and Parkinson’s diseases (PD) constitute a major public health problem due to an increasingly aged population as a consequence of generally improved medical care and demographic changes. However, current available treatments show modest symptomatic efficacy, leaving an unmet medical need for new, more effective therapies.

Staging AD and PD-associated neuropathological hallmarks has been essential to understand both disorders progression and to understand their pathological processes^1,2^. The pathological process in PD involves the intraneuronal accumulation of inclusion bodies, named Lewy bodies (LBs), composed of aggregates of α-synuclein (SNCA), ubiquitin and other proteins, found in the cytoplasm of neurons, often near the nucleus, or Lewy neuritis found in axons and dendrites^3,4^. Though, neurodegeneration occurs initially in the *substantia nigra pars compacta* (SNpc), a widespread neuronal loss in the other brain areas is also observed^1^. While the etiology of dopaminergic neuronal death is still difficult to understand mounting evidence implicate mitochondrial impairment and oxidative damage leading to axonal transport alterations and abnormal protein accumulation as key molecular mechanisms affecting the normal function of dopaminergic neurons^5^. The most prominent histopathological marks of AD are the presence of neurofibrillary tangles (NFTs), composed of filamentous aggregates called paired helical filaments (PHF) of hyperphosphorylated protein tau, frequently conjugated to ubiquitin, in cell bodies, neurophil threads in neuronal processes and neuritic (senile) plaques, which are extracellular deposits largely composed of fibrillar beta-amyloid (Aβ) peptides, usually seen in around dystrophic neurites^6,7^. AD is associated with neuronal loss, progressive synaptic and mitochondrial dysfunction, accompanied by the deposition of Aβ peptides and abnormal tau protein^7^. These hallmarks have been used as diagnostic criteria for the disease^2^, but whether they are causes of AD or merely consequences is yet unknown. In addition to the established pathology of senile plaques and neurofibrillary tangles, the presence of extensive oxidative stress is well characterized in AD brains^8^. Moreover, *post-mortem* studies in AD patients brains reveal disrupted mitochondrial *cristae*, reduced mitochondrial density and swelling, as well as, mitochondrial DNA (mtDNA) deletions^9^. In fact, accumulating evidence suggests that neuronal mitochondrial dysfunction affecting the energy supply of the cell, axonal traffic and autophagy induce AD characteristic synapse loss in the neocortex and hippocampus^10^. Vascular dementia (VD) is considered the second most common cause of cognitive impairment in the elderly population after AD with memory and cognitive function impairment as a consequence of reduced blood flow to the brain^11^. The heterogeneity of this disease makes it challenging to elucidate the underlying mechanisms and neuropathology. VD has been considered to be clinically distinctive from AD, however there are a number of similarities between these two disorders, specifically Aβ deposition^12^. Regarding VD pathophysiology, abnormal energy metabolism together with oxidative stress and reactive nitrogen species generation are known to contribute to cognitive impairments in VD culminating in hemodynamic abnormalities and neurovascular damage^13^.

Herein, we sought to follow the expression levels of protein markers implicated in microtubule assembly, autophagic-lysosomal pathway and unfolded protein response (UPR) in *post-mortem* samples from SNpc, hippocampus and temporal cortex from AD, PD and VD patients. The detected changes in these proteins markers enabled us to establish a time course for disease progression. Moreover, we will elucidate the underlying molecular mechanisms in light of the brain area, degree and extent of the disease and challenge the prion “like” SNCA and Aβ spreading hypothesis for PD^14,15^ and AD pathogenesis^16^.

## Results

### Parkinson’s disease

**Figure 1:**
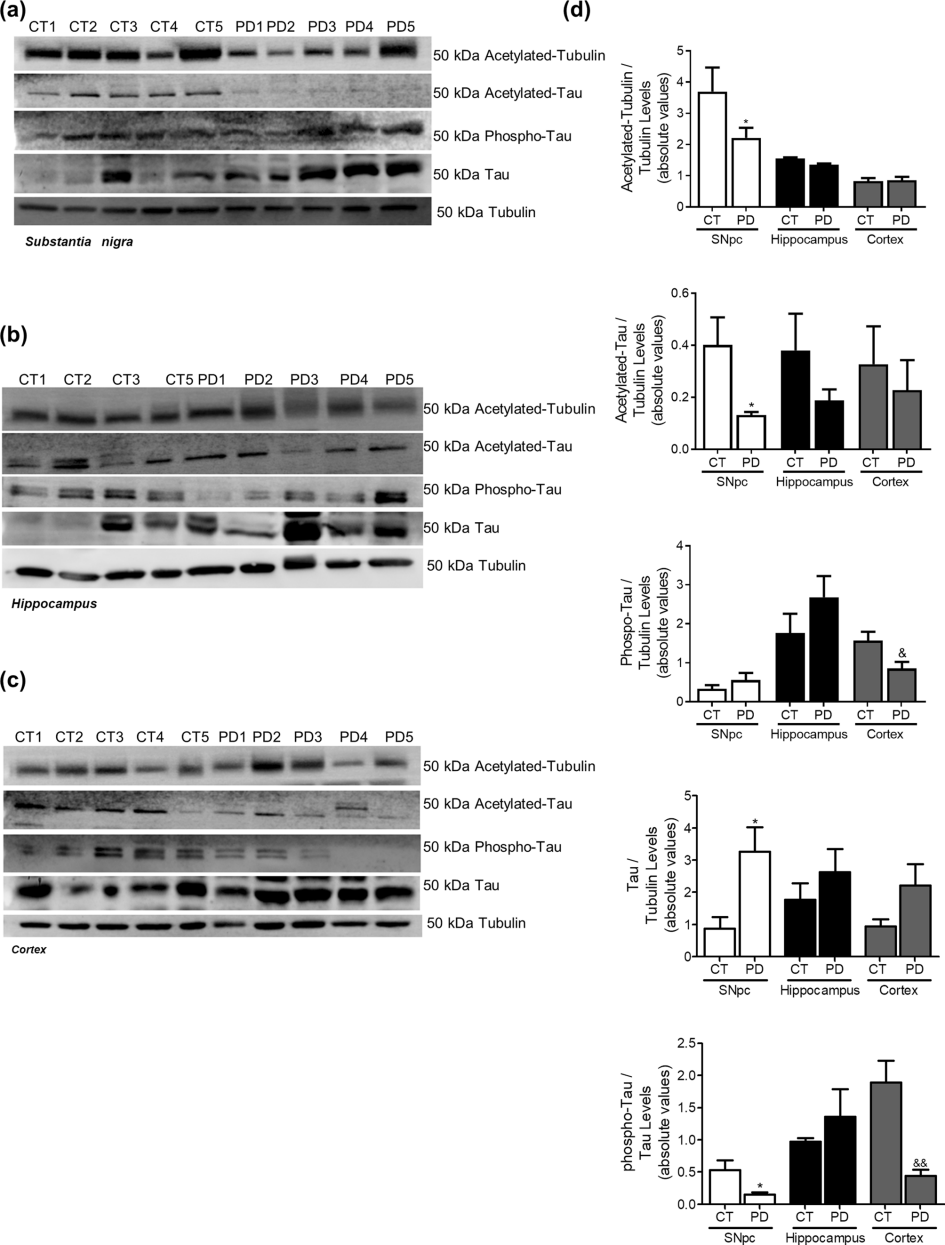
Microtubule Assembly in PD. Microtubule dynamics markers were determined in *post-mortem* human brain samples from SNpc, Hippocampus and Cortex of sporadic PD patients and controls. The levels of acetylated α-tubulin, acetylated tau, phosphor-tau and tau were determined in: (**A**) SNpc; (**B**) Hippocampus and (**C**) cortex brain tissue homogenates. (**D**) Densitometric analysis of the levels of acetylated α-tubulin, acetylated tau, phosphor-tau, tau and phosphor-tau/tau. The blots were re-probed for α-tubulin to confirm equal protein loading Values are mean ± SEM (n = 5, *p < 0.05, versus SNpc control subjects; ^&^p < 0.05 and ^&&^p < 0.01, versus cortex control subjects). Full length blots are presented in the Supplementary Information.

**Figure 2:**
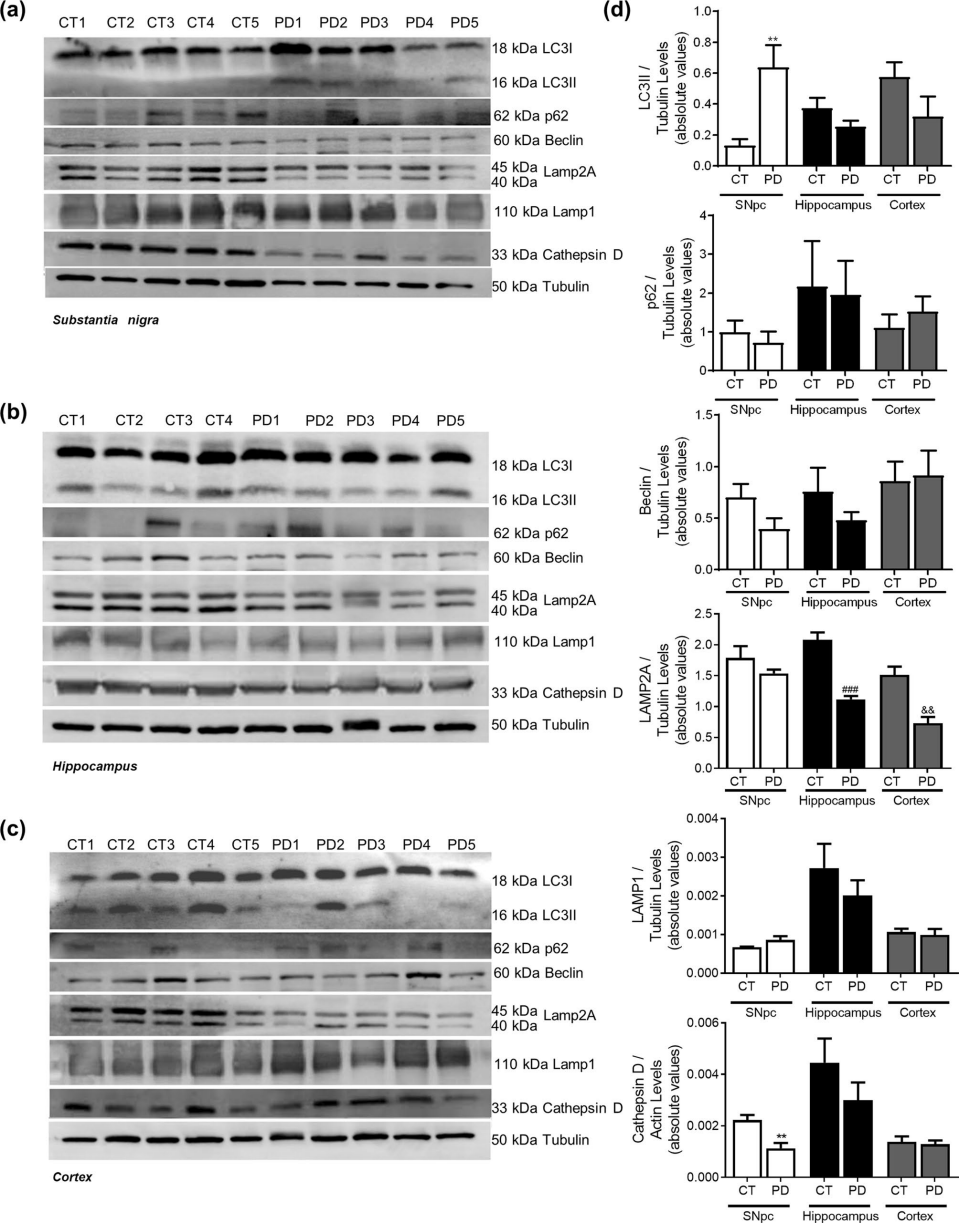
Autophagic-lysosomal pathway in PD. Autophagic and lysosomal markers were determined in *post-mortem* human brain samples from SNpc, Hippocampus and Cortex of sporadic PD patients and controls. The levels of LC3II, p62, Beclin1, Lamp2A, Lamp1 and CatD were determined in: (**A**) SNpc; (**B**) Hippocampus and (C) Cortex brain tissue homogenates. (**D**) Densitometric analysis of the levels of LC3II, p62, Beclin1, Lamp2A, Lamp1 and CatD. The blots were re-probed for α-tubulin to confirm equal protein loading Values are mean ± SEM (n = 5, **p < 0.01, versus SNpc control subjects; ^###^p < 0.001, versus hippocampus control subjects and ^&&^p < 0. 01, versus cortex control subjects). Full length blots are presented in the Supplementary Information.

**Figure 3:**
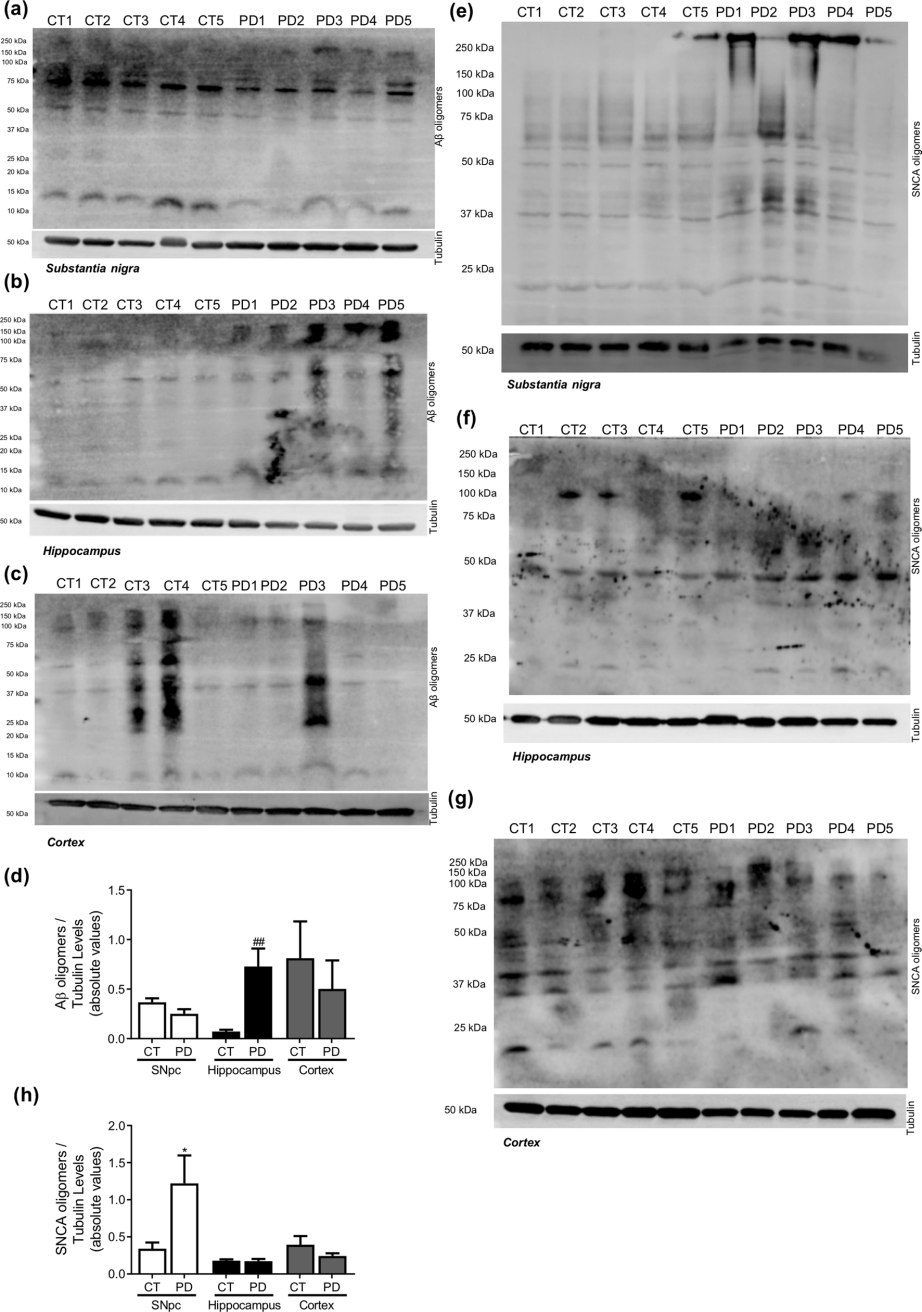
Neuropathological hallmarks in PD. Oligomeric proteins were determined in *post-mortem* human brain samples from SNpc, Hippocampus and Cortex of sporadic PD patients and controls. The levels of Aβ oligomers were determined in: (**A**) SNpc; (**B**) Hippocampus and (**C**) Cortex brain tissue homogenates. (**D**) Densitometric analysis of the levels of Aβ oligomers. The levels of SNCA oligomers were determined in: (**E**) SNpc; (**F**) Hippocampus and (**G**) Cortex brain tissue homogenates. (**H**) Densitometric analysis of the levels of SNCA oligomers. The blots were re-probed for α-tubulin to confirm equal protein loading Values are mean ± SEM (n = 5, *p < 0.05, versus SNpc control subjects; ^##^p < 0.01, versus hippocampus control subjects).

**Figure 4:**
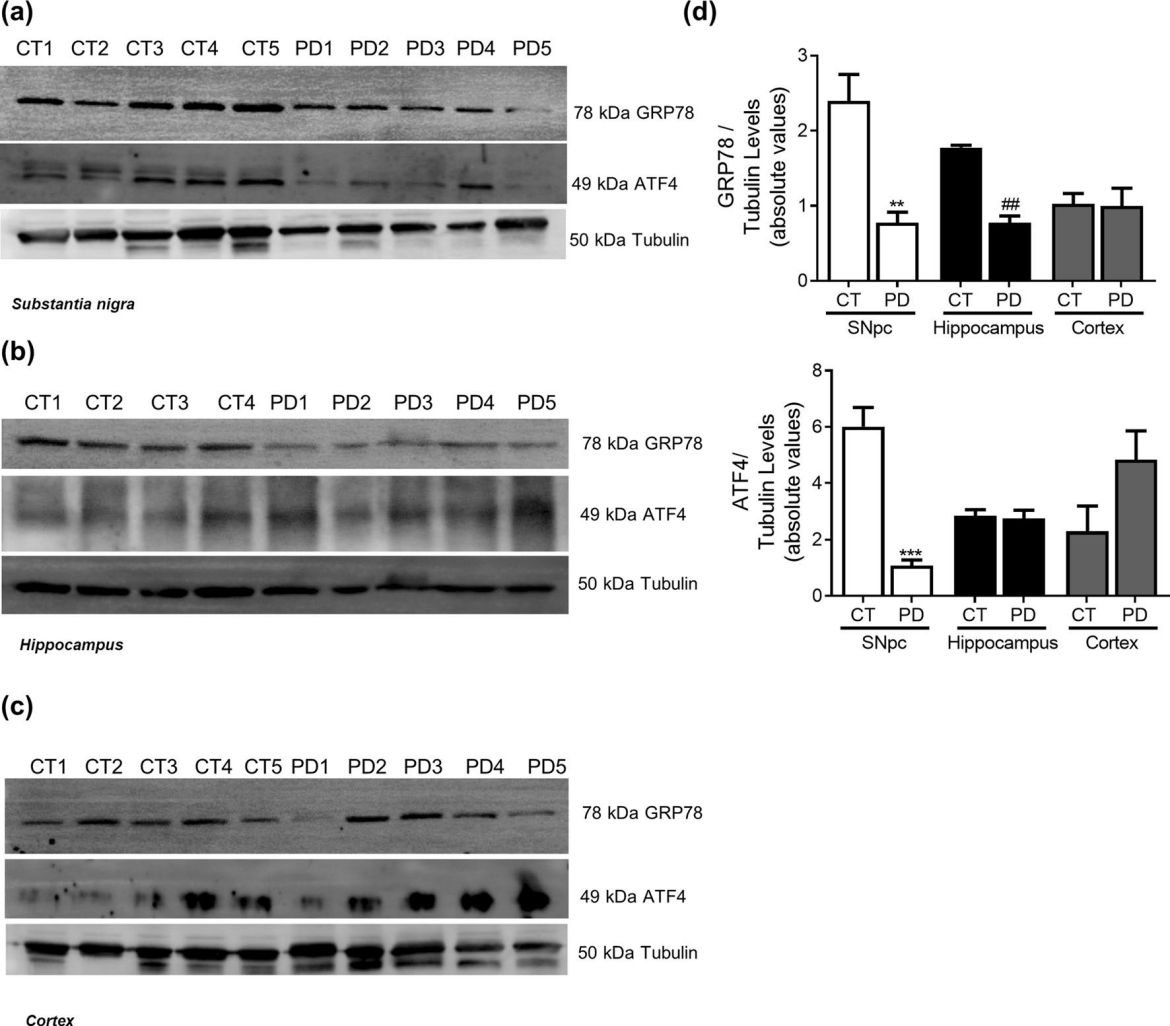
ER Stress in PD. UPR markers were determined in *post-mortem* human brain samples from SNpc, Hippocampus and Cortex of sporadic PD patients and controls. The levels of GRP78 and ATF4 were determined in: (**A**) SNpc; (**B**) Hippocampus and (**C**) Cortex brain tissue homogenates. (**D**). Densitometric analysis of the levels of GRP78 and ATF4. The blots were re-probed for α-tubulin to confirm equal protein loading Values are mean ± SEM (n = 5, **p < 0.01 and ***p < 0.001, versus SNpc control subjects; ^##^p < 0.01, versus hippocampus control subjects). Full length blots are presented in the Supplementary Information.

**Figure 5:**
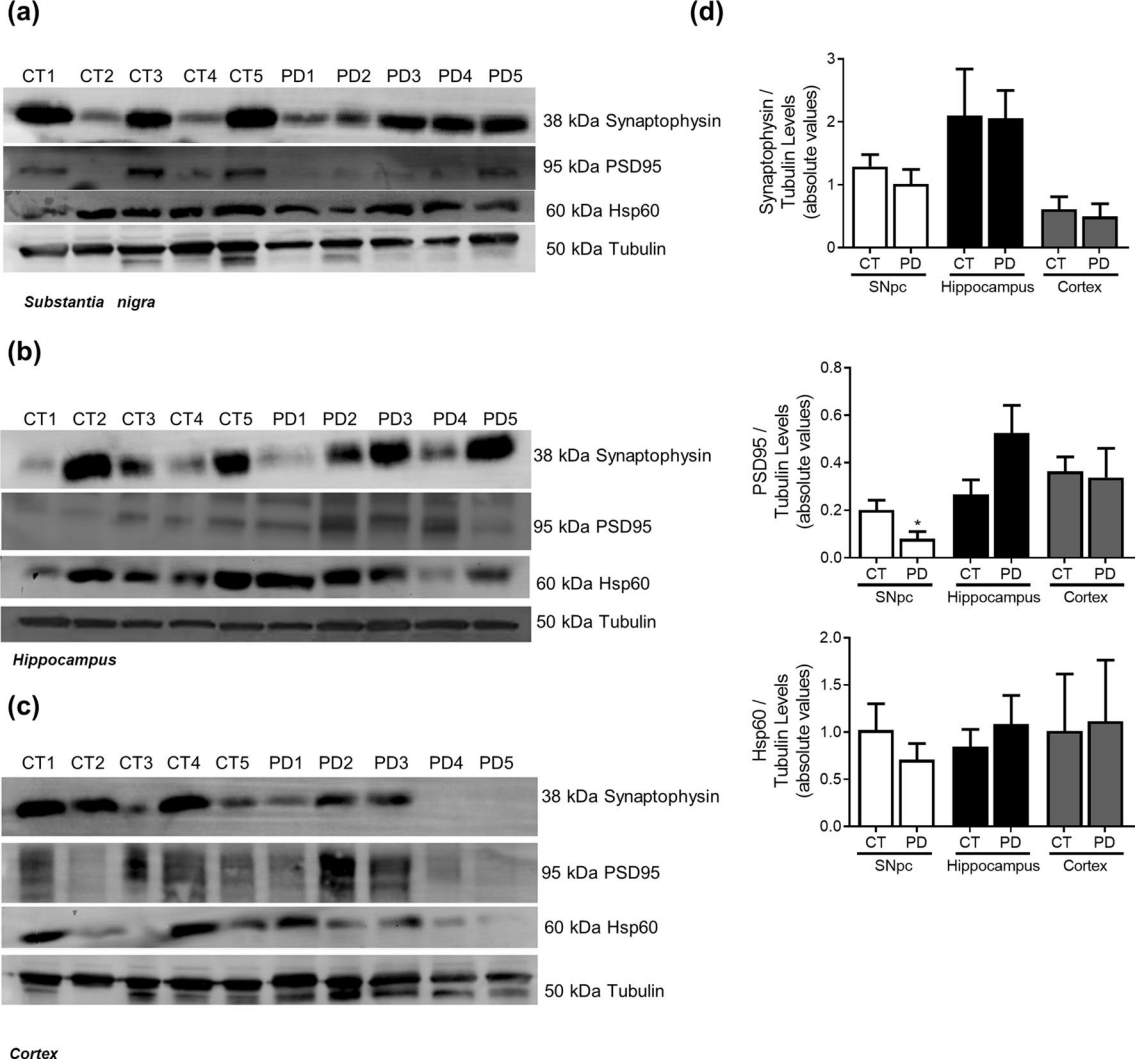
Synaptic markers in PD. Pre-synaptic, pos-synaptic and mitochondrial proteins were determined in *post-mortem* human brain samples from SNpc, Hippocampus and Cortex of sporadic PD patients and controls. The levels of synaptophysin, PSD95 and HSP60 were determined in: (**A**) SNpc; (**B**) Hippocampus and (**C**) Cortex brain tissue homogenates. (**D**) Densitometric analysis of the levels of GRP78 and ATF4. The blots were re-probed for α-tubulin to confirm equal protein loading Values are mean ± SEM (n = 5, *p < 0.05, versus SNpc control subjects). Full length blots are presented in the Supplementary Information.

We showed in PD cellular models that mitochondrial dysfunction triggers abnormal microtubule protein posttranslational modifications (PTMs), namely tubulin and tau acetylation and tau phosphorylation^10,17,18^. Additionally, it is well accepted that mitochondrial pools are dysfunctional in PD brain, namely in SNpc^19^ and also in peripheral cells^20,21^. In *post-mortem* human brain samples obtained from PD patients, Braak stage IV–VI, we observe a decrease in acetylated-tubulin and in acetylated-tau levels in SNpc (Fig. 1). These alterations were not evident in hippocampal or cortical samples indicating a specific effect on the brain structure more affected in PD. Interestingly, phospho-Tau levels were decreased in SNpc and cortical samples. Previous in vitro studies showed that microtubule disassembly induced by mitochondrial dysfunction impairs autophagy and decreases lysosomal activation leading to SNCA aggregation and neurodegeneration^17^. Herein, we observed an increase in LC3II levels and a decrease in Cathepsin D (CatD) levels (Fig. 2) in the SNpc. Unexpectedly, we found a decrease in LAMP2A levels in cortical and hippocampus samples. Correlated with autophagic alterations we found SNCA oligomers accumulation in the SNpc and most interestingly Aβ deposition in the hippocampus (Fig. 3). Taking into account that endoplasmic reticulum (ER) stress is a central contributor for proteostatic dysfunction we evaluated some key ER stress proteins^22^. We detected that the ER chaperone GRP78 and the transcription factor ATF4 involved in ER stress responses are decreased in PD SNpc (Fig. 4) indicating ER stress contribution to PD pathophysiology. Regarding synaptic markers, we only observed a decrease in the post-synaptic protein PSD95 in the SNpc (Fig. 5).

### Alzheimer’s disease

**Figure 6:**
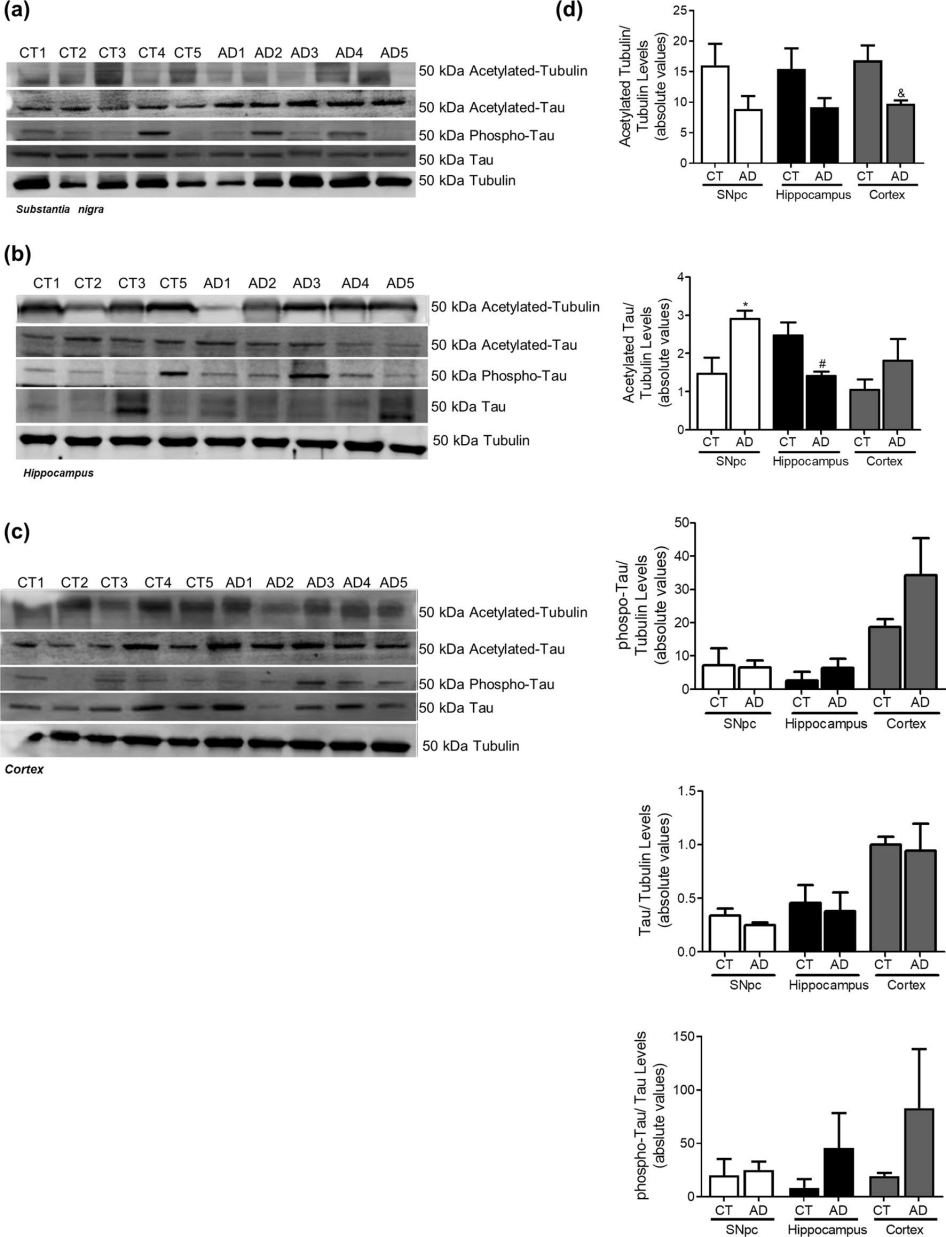
Microtubule Assembly in AD. Microtubule dynamics markers were determined in *post-mortem* human brain samples from SNpc, Hippocampus and Cortex of sporadic AD patients and controls. The levels of acetylated α-tubulin, acetylated tau, phosphor-tau and tau were determined in: (**A**) SNpc; (**B**) Hippocampus and (**C**) Cortex brain tissue homogenates. (**D**) Densitometric analysis of the levels of acetylated α-tubulin, acetylated tau, phosphor-tau, tau and phosphor-tau/tau. The blots were re-probed for α-tubulin to confirm equal protein loading Values are mean ± SEM (n = 5–6, *p < 0.05, versus SNpc control subjects; ^#^p < 0.05, versus hippocampus control subjects; ^&^p < 0.05, versus cortex control subjects). Full length blots are presented in the Supplementary Information.

**Figure 7:**
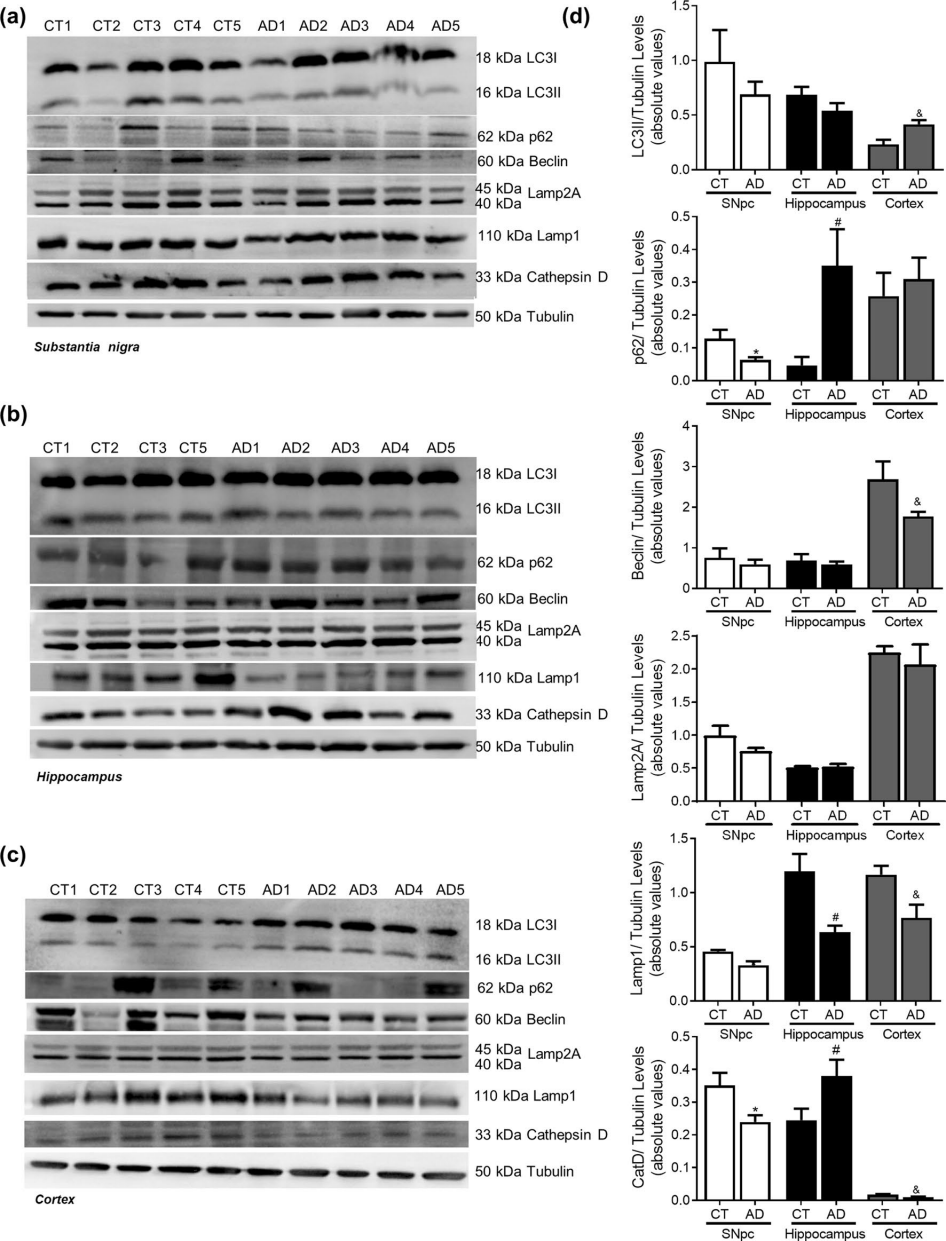
Autophagic-lysosomal pathway in AD. Autophagic and lysosomal markers were determined in *post-mortem* human brain samples from SNpc, Hippocampus and Cortex of sporadic AD patients and controls. The levels of LC3II, p62, Beclin1, Lamp2A, Lamp1 and CatD were determined in: (**A**) SNpc; (**B**) Hippocampus and (**C**) Cortex brain tissue homogenates. (**D**) Densitometric analysis of the levels of LC3II, p62, Beclin1, Lamp2A, Lamp1 and CatD. The blots were re-probed for α-tubulin to confirm equal protein loading Values are mean ± SEM (n = 5–6, *p < 0.05, versus SNpc control subjects; ^#^p < 0.05, versus hippocampus control subjects; and ^&^p < 0.05, versus cortex control subjects). Full length blots are presented in the Supplementary Information.

**Figure 8:**
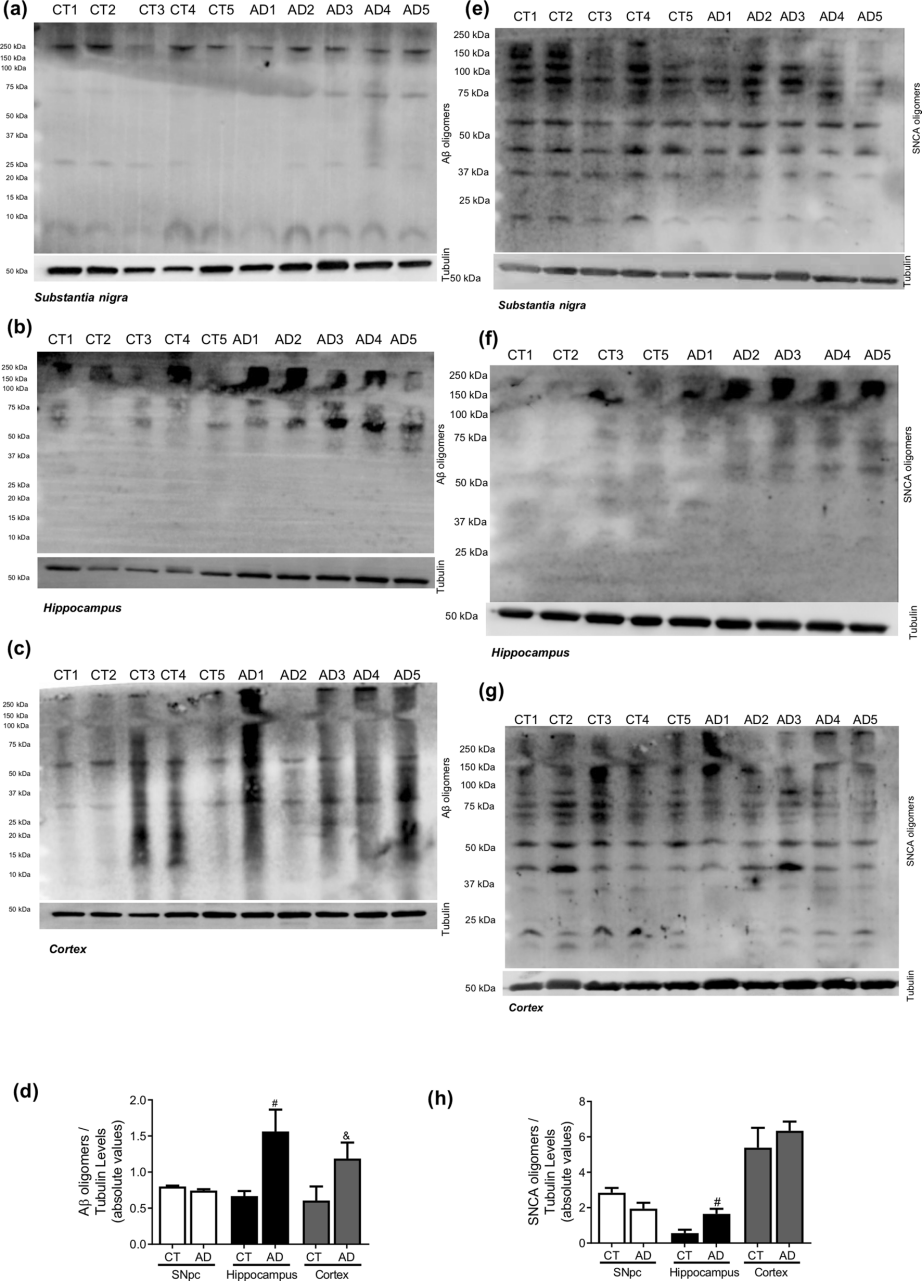
Neuropathological hallmarks in AD. Oligomeric proteins were determined in *post-mortem* human brain samples from SNpc, Hippocampus and Cortex of sporadic AD patients and controls. The levels of Aβ oligomers were determined in: (**A**) SNpc; (**B**) Hippocampus and (**C**) Cortex brain tissue homogenates. (**D**) Densitometric analysis of the levels of Aβ oligomers. The levels of SNCA oligomers were determined in: (**E**) SNpc; (**F**) Hippocampus and (**G**) Cortex brain tissue homogenates. (**H**) Densitometric analysis of the levels of SNCA oligomers. The blots were re-probed for α-tubulin to confirm equal protein loading Values are mean ± SEM (n = 5, ^#^p < 0.05, versus hippocampus control subjects; ^&^p < 0.05, versus cortex control subjects).

**Figure 9:**
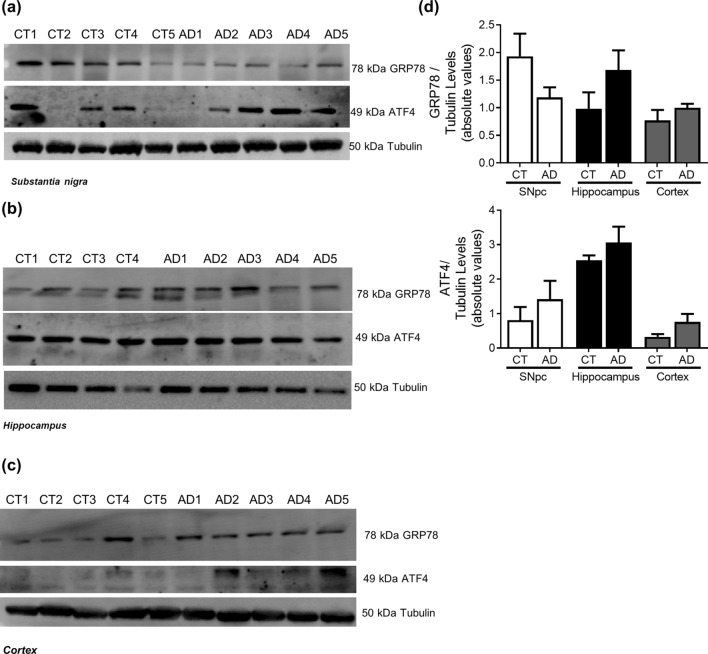
ER Stress in AD. UPR markers were determined in *post-mortem* human brain samples from SNpc, Hippocampus and Cortex of sporadic AD patients and controls. The levels of GRP78 and ATF4 were determined in: (**A**) SNpc; (**B**) Hippocampus and (**C**) Cortex brain tissue homogenates. (**D**) Densitometric analysis of the levels of GRP78 and ATF4. The blots were re-probed for α-tubulin to confirm equal protein loading Values are mean ± SEM (n = 5). Full length blots are presented in the Supplementary Information.

**Figure 10:**
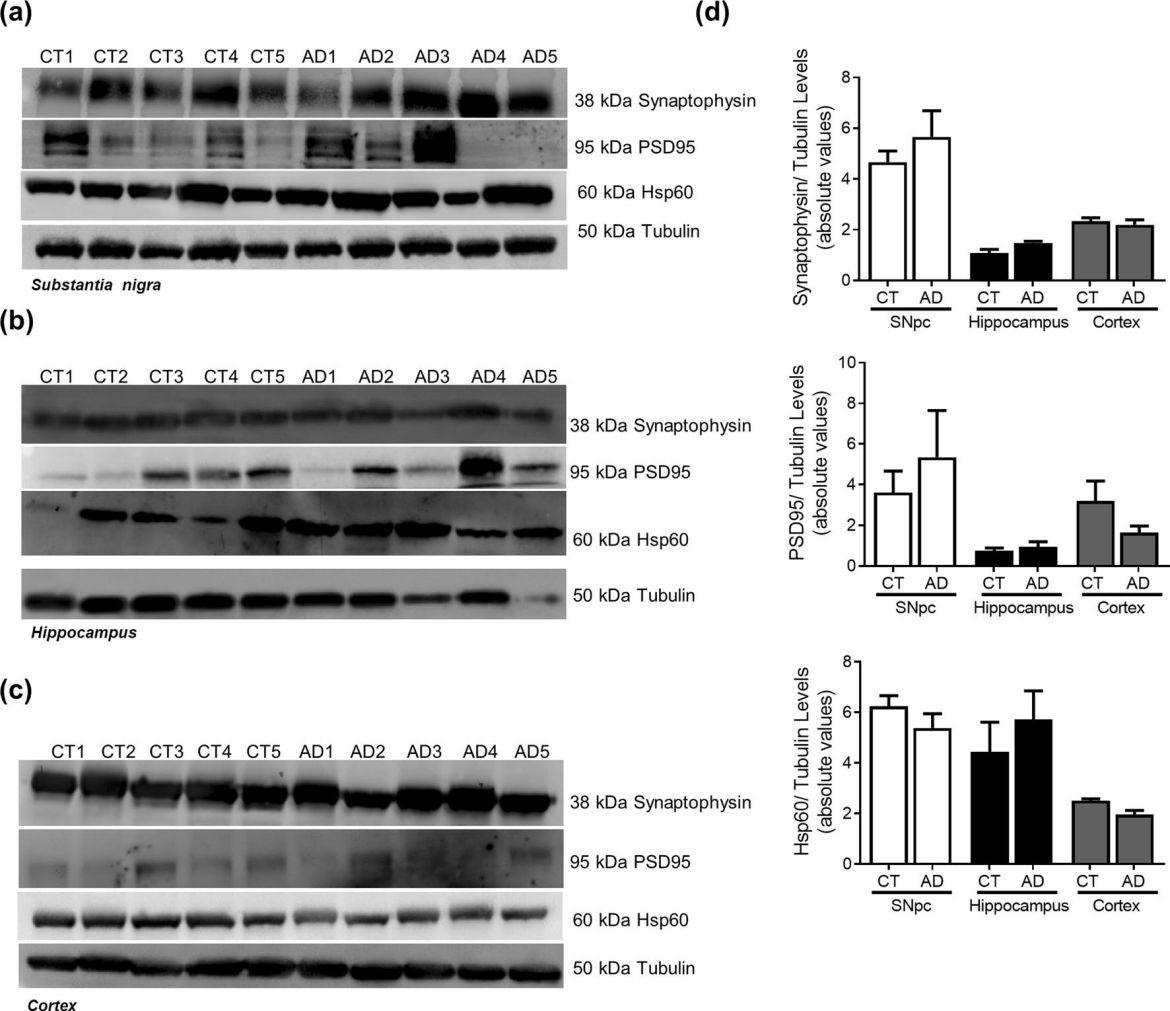
Synaptic markers in AD. Pre-synaptic, pos-synaptic and mitochondrial proteins were determined in *post-mortem* human brain samples from SNpc, Hippocampus and Cortex of sporadic AD patients and controls. The levels of synaptophysin, PSD95 and HSP60 were determined in: (**A**) SNpc; (**B**) Hippocampus and (**C**) Cortex brain tissue homogenates. (**D**) Densitometric analysis of the levels of GRP78 and ATF4. The blots were re-probed for α-tubulin to confirm equal protein loading Values are mean ± SEM (n = 5). Full length blots are presented in the Supplementary Information.

In AD cellular model studies we previously showed that dysfunctional mitochondria leading to alteration in PTMs modification, impacts microtubules assembly with a concomitant compromise of mitophagy and lysosomal activation,which increases Aβ production leading to hippocampal and cortical degeneration^10^. In human AD brain samples, Braak stage III–IV, analysed in this study, we observed a decrease in acetylated-tubulin and acetylated-tau levels, in accordance to an increase in phospho-Tau levels in the cortex and hippocampus (Fig. 6). Indeed, the expected alteration of microtubule assembly in AD-relevant brain areas may be responsible for the alteration observed in autophagic-lysosomal pathway. We observed an increase in LC3II and p62 levels in the cortex and hippocampus, a decrease in Beclin1 and CatD levels in the cortex and a decrease in Lamp1 levels in both areas (Fig. 7). As expected, we found Aβ buildup in AD cortical and hippocampal samples (Fig. 8). Remarkably, we also observed SNCA oligomerization in SNpc. ER stress has also been reported in AD^23^. However, we did not detect significant differences in the ER markers. Nonetheless, we observe a tendency to ATF4 and GRP78 increase in the cortex and in the hippocampus (Fig. 9). No differences were found for synaptic markers in AD samples (Fig. 10).

### Vascular dementia

**Figure 11:**
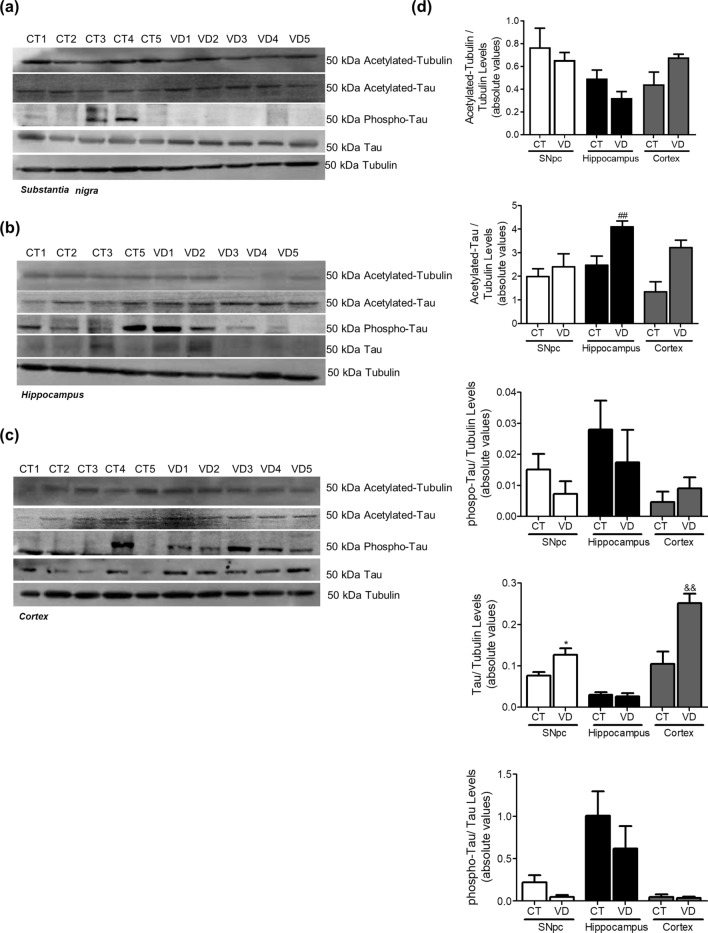
Microtubule Assembly in VD. Microtubule dynamics markers were determined in *post-mortem* human brain samples from SNpc, Hippocampus and Cortex of VD patients and controls. The levels of acetylated α-tubulin, acetylated tau, phosphor-tau and tau were determined in: (**A**) SNpc; (**B**) Hippocampus and (**C**) Cortex brain tissue homogenates. (**D**) Densitometric analysis of the levels of acetylated α-tubulin, acetylated tau, phosphor-tau, tau and phosphor-tau/tau. The blots were re-probed for α-tubulin ato confirm equal protein loading Values are mean ± SEM (n = 5–6, *p < 0.05, versus SNpc control subjects and ^##^p < 0.01, versus hippocampus control subjects; ^&&^p < 0.01, versus cortex control subjects). Full length blots are presented in the Supplementary Information.

**Figure 12:**
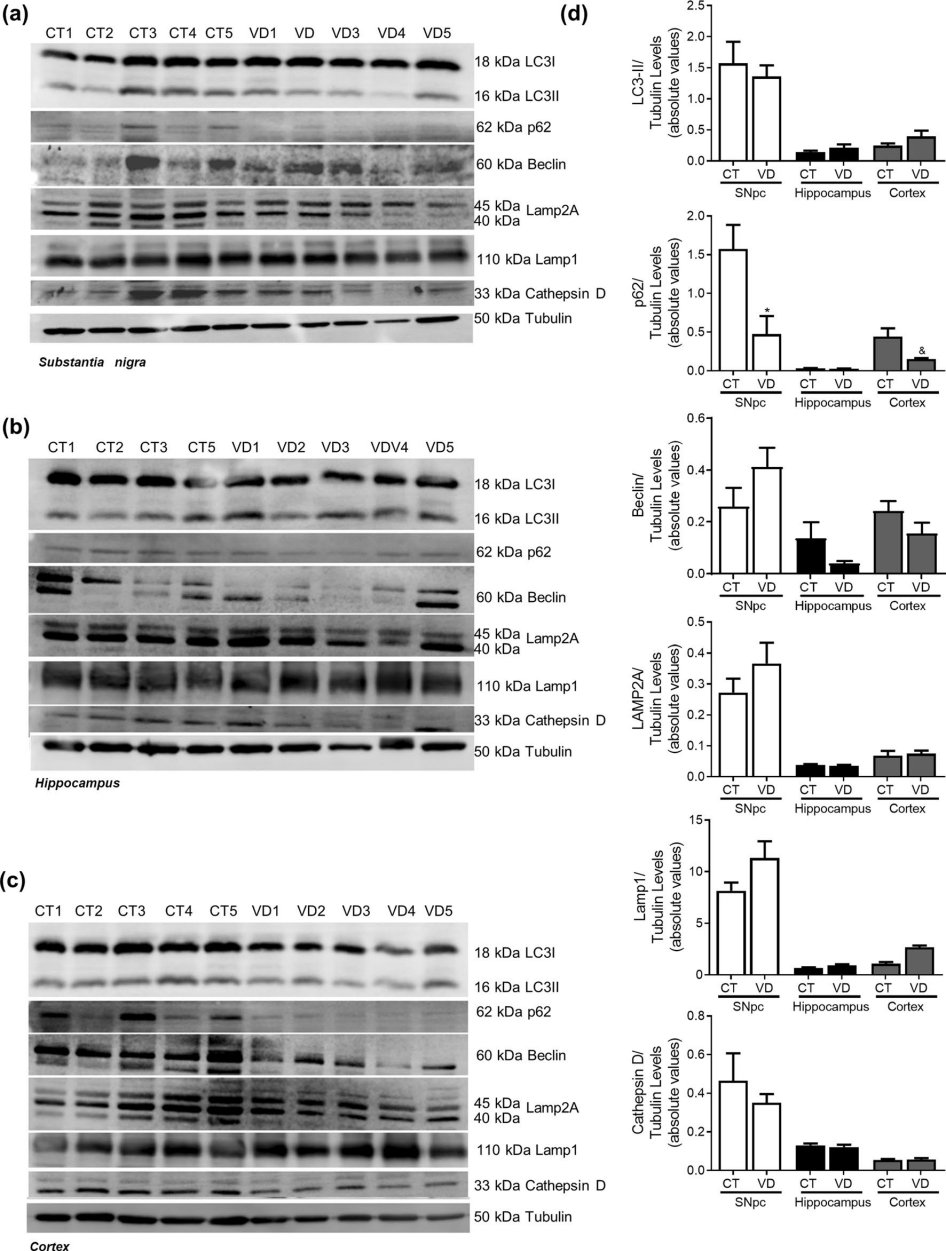
Autophagic-lysosomal pathway in VD. Autophagic and lysosomal markers were determined in *post-mortem* human brain samples from SNpc, Hippocampus and Cortex of VD patients and controls. The levels of LC3II, p62, Beclin1, Lamp2A, Lamp1 and CatD were determined in: (**A**) SNpc; (**B**) Hippocampus and (**C**) Cortex brain tissue homogenates. (**D**) Densitometric analysis of the levels of LC3II, p62, Beclin1, Lamp2A, Lamp1 and CatD. The blots were re-probed for α-tubulin to confirm equal protein loading Values are mean ± SEM (n = 5–6, *p < 0.05, versus SNpc control subjects and ^&^p < 0.05, versus cortex control subjects). Full length blots are presented in the Supplementary Information.

**Figure 13:**
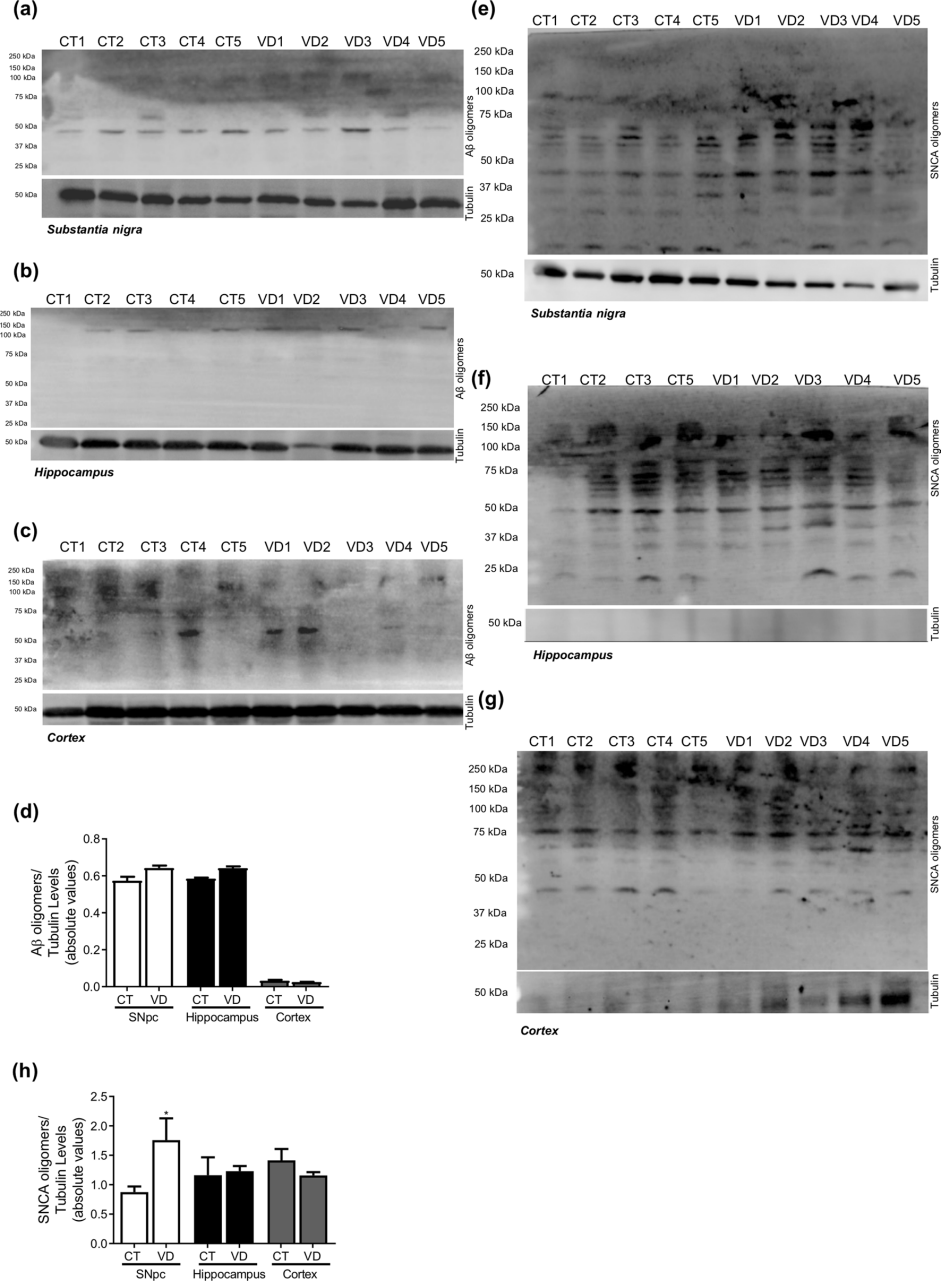
Neuropathological hallmarks in VD. Oligomeric proteins were determined in *post-mortem* human brain samples from SNpc, Hippocampus and Cortex of VD patients and controls. The levels of Aβ oligomers were determined in: (**A**) SNpc; (**B**) Hippocampus and (**C**) Cortex brain tissue homogenates. (**D**) Densitometric analysis of the levels of Aβ oligomers. The levels of SNCA oligomers were determined in: (**A**) SNpc; (**B**) Hippocampus and (**C**) Cortex brain tissue homogenates. (**D**) Densitometric analysis of the levels of SNCA oligomers. The blots were re-probed for α-tubulin to confirm equal protein loading Values are mean ± SEM (n = 5, *p < 0.05, versus SNpc control subjects).

**Figure 14:**
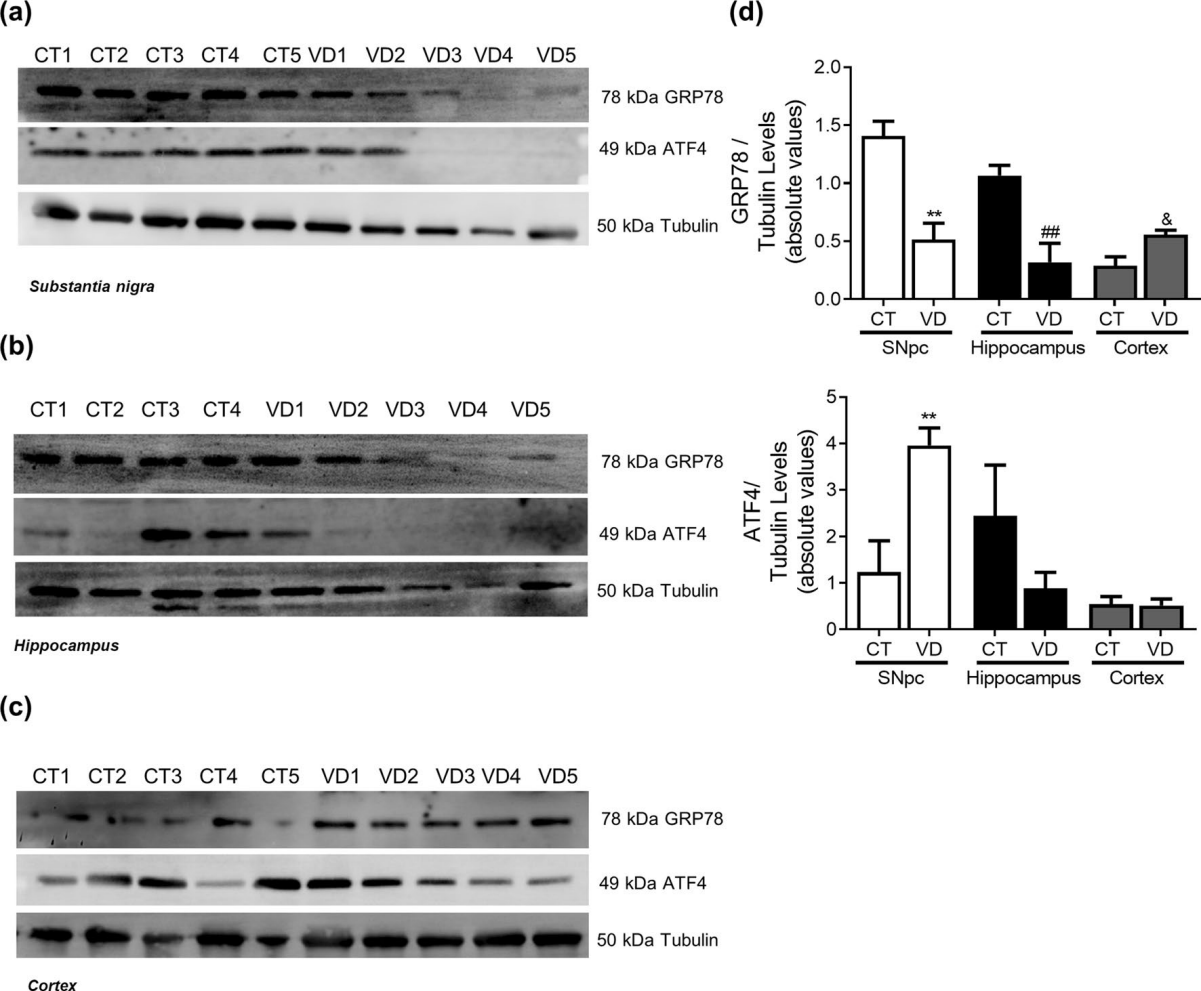
ER Stress in VD. UPR markers were determined in *post-mortem* human brain samples from SNpc, Hippocampus and Cortex of VD patients and controls. The levels of GRP78 and ATF4 were determined in: (**A**) SNpc; (**B**) Hippocampus and (**C**) Cortex brain tissue homogenates. (**D**) Densitometric analysis of the levels of GRP78 and ATF4. The blots were re-probed for α-tubulin to confirm equal protein loading Values are mean ± SEM (n = 5, **p < 0.01, versus SNpc control subjects; ^##^p < 0.01, versus hippocampus control subjects; ^&^p < 0.01, versus cortex control subjects). Full length blots are presented in the Supplementary Information.

**Figure 15:**
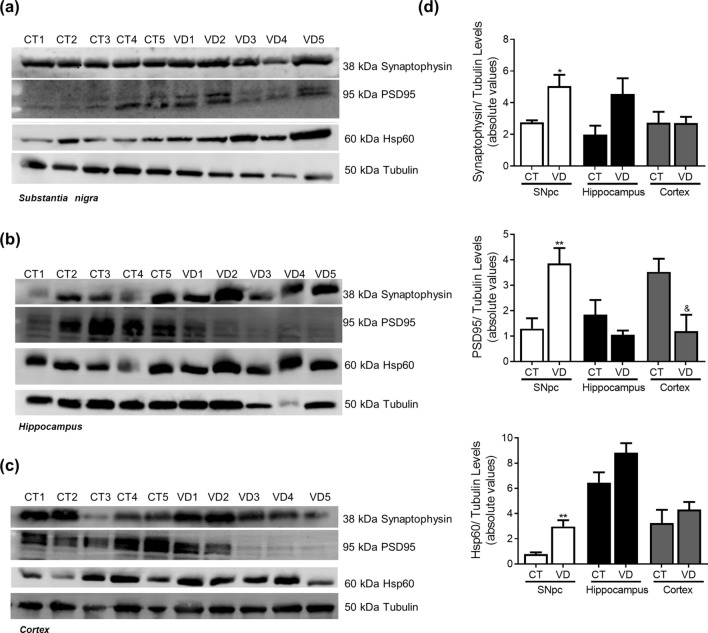
Synaptic markers in VD. Pre-synaptic, pos-synaptic and mitochondrial proteins were determined in *post-mortem* human brain samples from SNpc, Hippocampus and Cortex of VD patients and controls. The levels of synaptophysin, PSD95 and HSP60 were determined in: (**A**) SNpc; (**B**) Hippocampus and (**C**) Cortex brain tissue homogenates. (**D**) Densitometric analysis of the levels of GRP78 and ATF4. The blots were re-probed for α-tubulin to confirm equal protein loading Values are mean ± SEM (n = 5, *p < 0.05 and **p < 0.01, versus SNpc control subjects; ^&^p < 0.05, versus cortex control subjects). Full length blots are presented in the Supplementary Information.

The data obtained in brain areas of VD patients do not allow us to tackle a specific pathway, indeed we do not see any conclusive alterations in protein expression related to microtubule assembly, autophagic-lysosomal pathway and SNCA and Aβ oligomerization (Figs. 11, 12, 13). Nevertheless, this study indicates that ER stress may be a key event in VD neurodegeneration since we observed a decrease in GRP78 levels and an increase in ATF4 levels (Fig. 14). An interesting result was the fact that PSD95, synaptophysin and Hsp60 were augmented in the SNpc of VD samples (Fig. 15).

## Discussion

Using well categorized *post-mortem* brain tissues, this study is, to our knowledge, the first to comprehensively examine the differences between brain areas and cellular pathways affected in AD, PD.

We will discuss our results in light with the trigger, facilitator and aggravator hypothesis for PD, recently proposed and extending this premise to AD^24^. Indeed, this innovative hypothesis postulates that PD pathogenesis can be divided into three temporal phases. The first phase assumes that microbial infection, gut dysbiosis or environmental toxins ‘trigger’ the initiation of the disease process and are most prominent during the “prodromal PD” phase. Nevertheless, pathology progression needs ‘facilitators’ such peripheral inflammation or mitochondrial dysfunction. Indeed, it has been postulated that facilitators may promote the formation and/or seeding of pathogenic SNCA in the midbrain. After clinical onset we reach the third phase where ‘aggravators’ boost neurodegeneration and exacerbate symptoms. Johnson et al.^24^ proposed that impaired autophagy and cell-to-cell propagation of aggregated proteins (SNCA or eventually Aβ for AD) are the aggravators that lead to disease neurodegenerative progression in the brain. The human brain samples selected for this study with their Braak stage correspondence, IV–VI for PD cases and III–IV for AD cases, tell us that we are studying the aggravator phase of both disorders. Accordingly, we see an altered proteome in the SNpc of PD cases presenting a dysfunctional autophagic process, due to microtubule disassembly, altered levels of ER stress sensors and a decrease in post-synaptic markers. Our group previous work showed, in different PD models, that mitochondrial deficits lead to deficient intracellular traffic which results in incomplete autophagosome degradation and reduced autophagosome and mitochondrial movements culminating in a poor SNCA aggregate clearance^18,25,26^. Accordingly, we found that tau and tubulin acetylation are decreased resulting in microtubule disassembly and as consequence deficits in macroautophagy, seen by an accumulation of LC3II, which may potentiate SNCA accumulation in the SNpc. Indeed, PD cellular models show microtubule disassembly, due to tubulin depolymerization and deacetylation and tau hyperphosphorylation, postulating a direct involvement of tau in PD^18,26–28^. It was previously reported in sPD cybrids, in mtDNA-depleted cells and in cultured cells exposed to mitochondrial toxins such as 1-methyl-4-phenylpyridinium (MPP+), rotenone and 6-hydroxydopamine (6-OHDA) an augment in the number of autophagosomes^25,29,30^. Moreover, electron microscopy analysis revealed the autophagic vacuoles build-up in myelinized neurons of SNpc in PD patients^31^. Arduíno et al.^25^ also showed that induction of autophagy is not primarily affected since Beclin1 levels, a principal regulator in autophagosome formation, remained unaltered in sPD transmitochondrial cybrids relatively to CT cybrids. Likewise, we do not see any difference regarding Beclin1 levels. We posit that mitochondrial dysfunction could have played a role as disease facilitators, which contributed to the observed microtubule disassembly and impaired autophagy, a disease aggravator. It is widely known that SNCA enriched protein aggregates (LBs) are one of the main pathological hallmarks of PD. Interestingly, we did not found SNCA aggregates in the hippocampus or cortex of PD patients, which excludes the spread of SNCA pathology hypothesis^15^. Importantly, we observed that SNCA oligomerization is contained in SNpc, while we see lysosomal alterations, specifically LAMP2A levels reduction in the cortex and in the hippocampus and no changes in the SNpc indicating chaperone mediated autophagy alterations in these brain areas. Since in PD patients SNpc LAMP1 and LAMP2A levels are not altered but there is an increase in LC3II levels, we hypothesize that SNCA accumulation might be due to a deficient mobilization of autophagosomes from their site of formation toward lysosomes due to disruption in microtubule-dependent trafficking or due to an impairment of autophagosome–lysosome fusion. Remarkably LAMP2A expression decreased in other *post-mortem* PD brain samples^32^, which can be explained due to different Braak stages. Moreover, LAMP2A expression reduction was also observed in rats with targeted viral over-expression of SNCA^33^. Indeed, we found alteration in autophagic markers and Aβ oligomers build-up in the hippocampus of PD patients, which indicates progression of the neurodegenerative process that is not due to SNCA spreading. Several data collected from in vitro and in vivo models found evidence of a synergistic action between SNCA and Aβ^34,35^. In PD patients it was reported Aβ levels reduction in cerebrospinal fluid suggesting a possible contribution of Aβ not only on cognition but also on locomotor function^36^. Additionally, ER demise was found not only the SNpc but also in the hippocampus. Converging evidence reported the involvement of ER stress and the UPR in the pathophysiology of PD in in vivo and in vitro models of PD and in dopaminergic neurons in the SNpc of PD^37–40^. Our results lend strong support for ER and chaperone mediated autophagy demise in the hippocampus supporting hippocampus is also affected in PD late Braak stages.

With regard to the AD brain areas analysed in this study, and assuming that Braak stage III–IV fits on the aggravator phase of the disease, we observe relevant alterations in key autophagic proteins, such as increase in LC3II and p62 levels in the hippocampus and cortex, indicating that autophagosomes are being formed but accumulate probably due to a failure in lysosomal function, since we also saw a decrease in Lamp1 levels. In fact, the involvement of macroautophagy in AD neurodegenerative process was shown in neocortical biopsies from AD brain where immature autophagic vacuoles were abundant within dystrophic neurites^41^. Additionally, work from our group showed that in differentiated sAD patient-driven cells, autophagosomes transport velocity is decreased, therefore will not reach lysosomes for degradation of their contents contributing to a compromised autophagic flux^10^. We found a decrease in CatD levels in cortical samples but an increase in the hippocampus. There is some controversy regarding lysosomal enzymes levels in association with AD. Whereas CatD has been reported to be decreased in skin fibroblasts from patients with AD^42^ and in the hippocampus of the PS1M146L/APP751sl mouse model^43^, this lysosomal enzyme has been shown to be elevated in the CSF in patients with AD^44^. Chai et al.^45^ also found in neocortical regions of well characterized AD brains elevated CatD immunoreactivity. Additionally, the observed decrease in Lamp1 levels in the hippocampus and cortex of AD patients, corroborates our hypothesis that the lysosomal function may be affected. Nevertheless, Lamp1 was shown to be elevated in brain-derived blood exosomes of patients with AD compared with controls^46^. We also observed a reduction in Beclin1 levels in AD susceptible areas, namely the hippocampus and cortex. Indeed, Beclin1 expression was found to be reduced in AD cybrids, as well as, in AD brain samples indicating that the initial steps of this pathway may be compromised^10,47^. These alterations in autophagic markers correlate with the increase in Aβ oligomer levels, which is a key histopathologic feature of AD^48^. In this context, we also see alterations in PTMs in microtubule proteins, namely an increase in phospho-tau levels. In transgenic AD mice cytoskeletal defects were shown to be correlated with the build-up of filled autophagic vacuoles in dystrophic neurites^49^. Furthermore, in AD cellular models tubulin acetylation was found to be reduced together with an increase of phospho and acetylated-tau. This pathway leads to microtubule disassembly which compromises autophagic vacuoles transport as observed by a decrease in the rate of LC3II degradation^18,50^. Also, confirmation from AD brain homogenates point to microtubule disassembly related with mitochondrial abnormalities before detectable NFT^51^. Neuronal microtubules were found to be lessened in number and length^52^, acetylated-tubulin levels were diminished and tau phosphorylation was significantly increased^10,53,54^. The selected AD cases were in mild-moderate phase (Braak stage III–IV) of the disease and so, we still detect a tendency for an activation of ER UPR. Cortical neurons exposed to Aβ show ER Ca2+ stores depletion and increased levels of ER stress markers such as GRP78 leading to the activation of an ER-mediated apoptotic cell death^55^. Furthermore, GRP78 has been shown to be increased in AD *post-mortem* brain tissues, demonstrating that sustained ER stress is involved in neurodegeneration^56^. In AD transgenic mice ER stress-related genes are differentially regulated during Aβ deposition in the hippocampus and cortex^57^. On the other hand, in 5xFAD AD mouse model that displays aggressive amyloid pathology no elevation in the ER stress markers was found^58^. Again, our results do not support a relevant role for the propagation of Aβ oligomers to other brain areas^16^ since we show alteration in autophagy in SNpc of AD patients, proposed to be involved in the aggravator phase, but no increase in Aβ aggregates. Nonetheless, we do observe that SNCA oligomers are present in the hippocampus of AD cases, which may indicate that this event is neuronal specific. Indeed, in a mouse model of tauopathy immunohistochemistry studies showed accumulation of not only hyperphosphorylated tau but also aggregates of phosphorylated SNCA^59^. Interestingly, SNCA is also implicated in AD pathogenesis. SNCA staining was found to be elevated in AD brain when compared to controls and as well in Tg2576 AD mice brain model^60^. Additionally, it has been conjectured that SNCA is involved in the abnormal synapse formation in AD brain patients^61^.

**Figure 16:**
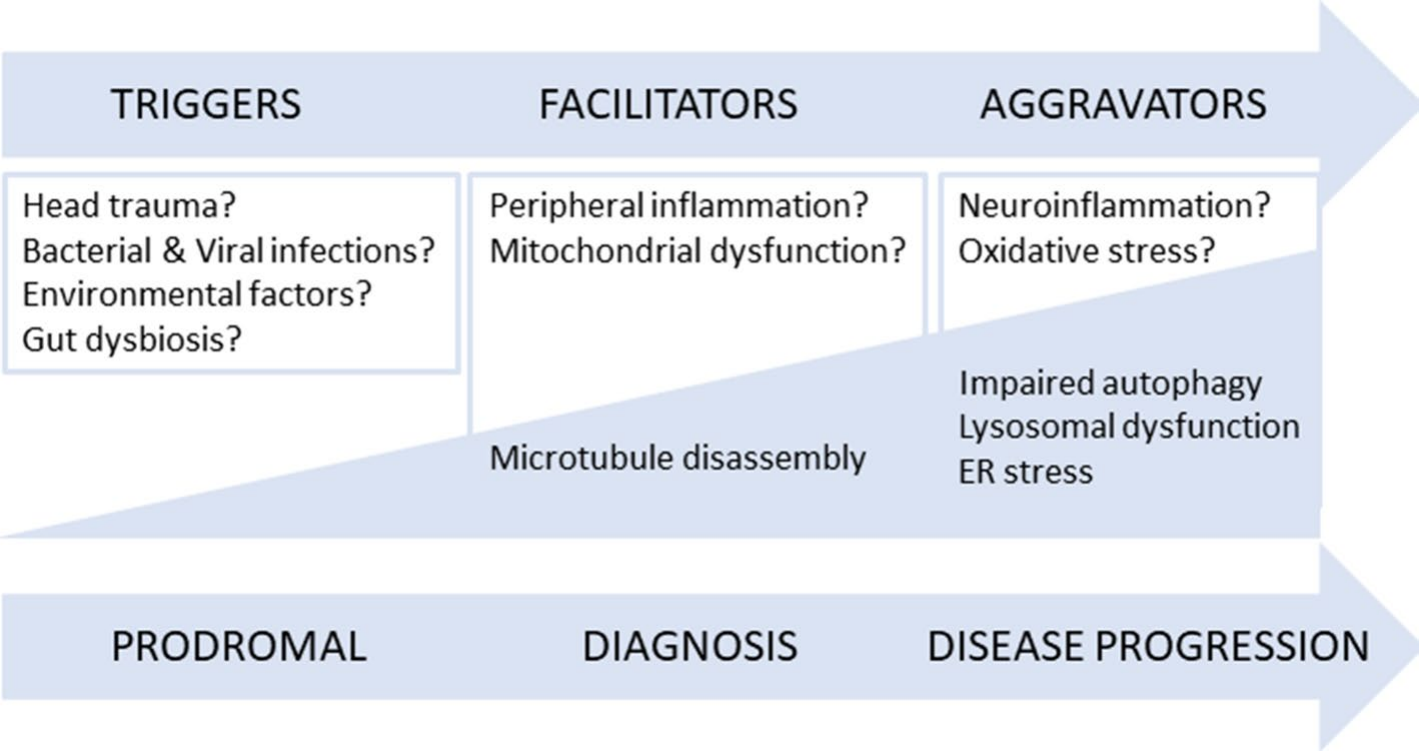
Revised hypothesis of the ‘triggers, facilitators and aggravators’ for PD and AD pathogenesis. Our results are in accordance with the hypothesis proposed by Johnson et al.^24^ and can be partially applied to both PD and AD pathogenesis. Our data obtained from human post-mortem brain samples staged in the aggravator phase show that autophagy is impaired in key areas, but we do not confirm cell-to-cell propagation of aggregated proteins, since SNCA aggregates only appear in the SNpc of PD patients and Aβ aggregates are only present in the hippocampus and cortex of AD patients. Our data also support that microtubule disassembly due to mitochondrial dysfunction, a proposed facilitator, prompts the onset of PD or AD symptoms and might trigger autophagic deficits that will exacerbate cell loss and neuropathology.

As a control we used the same brain areas of VD cases to determine the specificity of the pathways altered in AD and PD. Accumulating evidence show that a large proportion of patients with dementia who have significant cerebral vascular lesions also exhibit more severe concomitant AD pathology^62^, such as deposits of hyperphosphorylated-tau and Aβ, and thus fulfil the neuropathological criteria for AD. Indeed, we found an increase in tau levels in the SNpc and in acetylated tau in the hippocampus. A report from 2015 showed a selective loss of total tau in the temporal cortex of VD patients relative to controls whereas phosphorylated tau levels remained unchanged in VD patients^63^. Furthermore, VD brain blood barrier dysfunction and altered cerebrovascular permeability can result in overproduction of Aβ and oxidative stress^64^. Although elevated vascular risk may influence tau burden when coupled with high Aβ burden we do not see Aβ accumulation in VD patients^65^. Remarkably, we observed SNCA over-production in SNpc VD samples. Indeed, a clinicopathological study found no evidence for increased SNCA deposition in subjects with brain vascular pathological changes^66^. We did not see any conclusive or consistent alteration in protein expression related to autophagic-lysosomal pathway. Still, autophagy enhancement was found in animal models of VD^67^. It is also worth noting that this study indicates that ER stress may be a key event in VD neurodegenerative process. In fact, in a neuronal model of VD low concentrations of copper potentiate zinc-induced ER stress pathways^68^. Moreover, DL-3-n-butylphthalide was shown to protect against cognitive deficits in a rat model of VD by regulating ER stress-related markers^69^. This work highlights a new conceptual model for AD and PD pathogenesis. Taking this into account we analyzed the aggravator phase in AD and PD *post-mortem* samples and provided an updated summary of the cellular pathways and proteins involved in the pathophysiology of these neurodegenerative disorders. Of particular importance is the fact that the aggravator phase in the development of these diseases also includes microtubule-dependent transport defects that result from mitochondrial dysfunction and contribute to autophagic alterations culminating in protein aggregation. Another focus of potential significance is that Aβ accumulation was found in *post-mortem* PD hippocampus and SNCA accumulation was found in *post-mortem* AD hippocampus and SNpc. These results indicate an involvement of SNCA in AD and of Aβ in PD, at least in the moderate to late Braak stages. Consistently, a common feature in these disorders is protein aggregation, but our results clearly contest the prion “like” propagation hypothesis (revised in^15^), since SNCA oligomers are contained to SNpc in PD samples and Aβ aggregates only occur in the cortex and hippocampus in AD samples (revised in^16^). In summary, the aforementioned results raise the possibility that the hypothesis “triggers, facilitators and aggravators” proposed by Johnson et al.^24^ can be partially applied to both PD and AD pathogenesis (Fig. 16). Overall, we posit that future studies need to focus on the fact that effective treatments must be disease stage specific.

## Material and methods

### Human tissue

Human brain tissue was obtained from the Neurological Tissue Bank, Biobanc-HospitalClinic-IDIBAPS and as generous gift from Professor I Ferrer Abizanda, Bellvitge Hospital Universitari, Institut Català de la Salut, Barcelona, Spain and used under local regional ethical approval (Law 14/2007 on Biomedical Research). Frozen samples of SNpc, Hippocampus and Temporal Cortex from idiopathic PD patients (n = 5), idiopathic AD patients (n = 5), VD patients (n = 5) and age- and gender-matched controls (n = 6) were used. All patients had been diagnosed according to the diagnostic criteria of the Neurological Tissue Bank. The neuropathological assessments of disease cases were performed and Braak neuropathological staging found AD patients to be at Braak stages 3–4 and PD patients to be at Braak stages 4–5–6. None of the patients were believed to have alternative diagnoses, degeneration of related systems, drug induced Parkinsonism, or any other serious medical illness. Enrolment was also contingent on the absence of a diagnosis for another neurodegenerative disease. The control subjects have not been diagnosed with a neurodegenerative or pre-neurodegenerative disease condition. The gender, age at death, and clinical diagnosis of the study cohort are summarized in Table 1.

**Table 1:** Demographic and medical information of case studies.

Human brain samples	Gender	Age	Diagnosis
Control 1	Male	73	Acute pancreatitis
Control 2	Female	73	Metabolic syndrome
Control 3	Male	70	Pancreatic metastatic adenocarcinoma
Control 4	Male	76	Cardiac failure
Control 5	Male	63	Myeloma
Control 6	Female	56	Pulmonary oat carcinoma
PD 1	Female	81	Braak stage V-PD
PD 2	Male	81	Braak stage V/VI-PD
PD 3	Male	77	Braak stage V-PD
PD 4	Male	77	Braak stage IV/V-PD
PD 5	Male	74	Braak stage V-PD
AD 1	Male	81	Braak stage III-AD
AD 2	Male	84	Braak stage IV-AD
AD 3	Male	89	Braak stage IV-AD
AD 4	Male	74	Braak stage III-AD
AD 5	Male	88	Braak stage III-AD
VD 1	Male	78	Vascular dementia
VD 2	Female	73	Vascular dementia
VD 3	Male	80	Vascular dementia
VD 4	Female	74	Vascular dementia
VD 5	Female	85	Vascular dementia

### Preparation of human brain samples

Brain tissue was preserved at − 80 °C, and a small fraction of tissue (1 mg) was homogenized in a glass homogenizer in 1% Triton X-100 containing hypotonic lysis buffer (25 mM HEPES, 2 mM MgCl_2_, 1 mM EDTA and 1 mM EGTA, pH 7.5) supplemented with 2 mM DTT, 0.1 mM PMSF, and a 1:1,000 dilution of a protease inhibitor cocktail from Sigma (St. Louis, MO, USA). Tissue suspensions were then frozen three times in liquid nitrogen and centrifuged at 20,000×*g* for 10 min. The resulting supernatants were removed and stored at − 80 °C^70^. Protein content of brain homogenates was determined using Pierce™ BCA Protein Assay Kit (Thermo Scientific, Rockford, IL, USA) according to the manufacturer’s instructions for plate reader.

### Western blotting analysis

Western blotting analysis was performed as previously described in^71^ with slight modifications. For the analysis of Aβ and SNCA oligomers, samples were loaded under non-reducing and non-denaturating conditions. For the analysis of the remaining proteins, the samples were resuspended in 6 × sample buffer (4 × Tris–Cl/SDS, pH 6.8, 30% glycerol, 10% SDS, 0.6 M DTT, 0.012% bromophenol blue) under reducing conditions. Depending on the protein molecular weight, samples were loaded onto accordingly % SDS-PAGE gels. After transfer to PVDF membranes (Millipore, Billerica, MA, USA), the membranes were incubated for 1 h in Tris-buffered solution (TBS) containing 0.1% Tween 20 and 5% BSA, followed by an overnight incubation with the respective primary antibodies at 4 °C with gentle agitation. The primary antibodies used were the following: 1:1,000 polyclonal anti-alpha-SNCA, oligomer specific Syn-33 from Sigma (St. Louis, MO, USA) (cat number: ABN2265); 1:1,000 polyclonal anti-LC3B (microtubule-associated protein 1A/1B-light chain 3) from Cell Signaling (Danvers, MA, USA) (cat number: 3868); 1:16,000 monoclonal anti-acetylated α-tubulin from Sigma (St. Louis, MO, USA) (cat number: T7451); 1:1,000 monoclonal anti-synaptophysin from Sigma (St. Louis, MO, USA) (cat number: S5768); 1:1,000 anti-PSD95 (postsynaptic density protein 95) antibody from Abcam (Cambridge; UK) (cat number: ab18258); 1:1,000 anti-Lamp2A (lysosomal-associated membrane protein 2A) from Novus Biologicals (Littleton, CO, USA) (cat number: ab37024); 1:750 anti-Tau Acetyl K280 from AnaSpec (Fremont, CA, USA) (cat number: AS-56077); 1:1,000 anti-Beclin1 from Cell Signaling (Danvers, MA, USA) (cat number: 3738); 1:1,000 anti-p62 from Sigma (St. Louis, MO, USA) (cat number: P0067); 1:1,000 anti-Lamp1 (lysosomal-associated membrane protein 1) from Cell Signaling (Danvers, MA, USA) (cat number: 3243); 1:750 anti-phospho-Tau Ser396 from Santa Cruz Biotechnology (Santa Cruz, CA, USA) (cat number: sc-101815); 1:1,000 anti-Aβ from Cell Signaling (Danvers, MA, USA) (cat number: 8243); 1:1,000 anti-cathepsin D (CatD) from Santa Cruz Biotechnology (Santa Cruz, CA, USA) (cat number: sc-377124); 1:1,000 anti-Hsp60 from BD Transduction Laboratories (San Jose, CA, USA) (cat number: 611563); 1:1,000 monoclonal anti-Tau, clone Tau46 from Sigma (St. Louis, MO, USA) (cat number: T9450); 1:1,000 anti-BiP/GRP78 (78-kDa Glucose-regulated protein) from BD Transduction Laboratories (San Jose, CA, USA) (cat number: 610979); 1:1,000 anti-ATF4 (Activating transcription factor 4) from Cell Signaling (Danvers, MA, USA) (cat number: 97038). Monoclonal anti-α-tubulin (1:10,000) from Sigma (St. Louis, MO, USA) (cat number: T 6199) was used for loading control. Human brain alpha-synuclein was recognized by mass spectrometric detection. Membranes were washed with TBS containing 0.1% non-fat milk and 0.1% Tween three times (each time for 10 min), and then incubated with the appropriate horseradish peroxidase-conjugated secondary antibody for 2 h at RT with gentle agitation. After three washes, specific bands of interest were detected by developing with an alkaline phosphatase enhanced chemical fluorescence reagent (ECF from GE Healthcare, Piscataway, NJ, USA). Fluorescence signals were detected using a Biorad Versa-Doc Imager, and band densities were determined using Quantity One Software. Membranes were revealed using Biorad Versa-Doc Imager. Quantitative densitometric analysis was performed using Quantity One Software and expressed relative to alpha-tubulin. Full length blots are presented in Supplementary Information.

### Statistical analysis

Statistical analyses were performed using PRISM 5 (GraphPad Software, San Diego, CA, USA). All data were expressed as group means ± SEM of at least four different samples. Differences between two datasets were evaluated by two-tailed unpaired Student’s t test. Statistical tests between multiple datasets and conditions were carried out using a one-way analysis of variance (ANOVA) followed by Bonferroni post hoc test to determine statistical significance, as appropriate. A p value < 0.05 was considered statistically significant.

### Equipment and settings section

Touch-up tools in Photoshop were not used. Excessive processing and manipulation of images was not performed.

### Ethical standards

We confirm that all methods were carried out in accordance with relevant guidelines and regulations. We confirm that all experimental protocols were approved by our institute, the Center of Neuroscience and Cell Biology. We used *post-mortem* brain samples from Parkinson’s, Alzheimer’s disease, Vascular Dementia patients and age-matched controls. Brain samples were acquired from the Neurological Tissue Bank Biobanc-Hospital Clinic-IDIBAPS in Spain. Brain tissue is fully anonymized and the Biobank is operating accordingly with European laws. Informed consent was obtained from all subjects or, legal guardian. Faculty of Medicine, University of Coimbra Ethical Committee approved our request to use human brain samples.

### Data availability

All data generated or analysed during this study are included in this published article (and its Supplementary Information files).

## Supplementary information

Supplementary Information.

## Abbreviations

MPP+: 1-Methyl-4-phenylpyridinium
6-OHDA: 6-Hydroxydopamine
GRP78: 78-KDa glucose-regulated protein
ATF4: Activating transcription factor 4
AD: Alzheimer’s disease
Aβ: Beta-amyloid
CatD: Cathepsin D
ER: Endoplasmic reticulum
LBs: Lewy bodies
Lamp 1: Lysosomal-associated membrane protein 1
Lamp 2A: Lysosome-associated membrane protein 2A
LC3: Microtubule-associated protein 1A/1B-light chain 3
mtDNA: Mitochondrial DNA
NFTs: Neurofibrillary tangles
PHF: Paired helical filaments
PD: Parkinson’s disease
PSD95: Postsynaptic density protein 95
PTMs: Posttranslational modifications
SNpc: Substantia nigra pars compacta
UPR: Unfolded protein response
VD: Vascular dementia
SNCA: α-Synuclein
