# Supplementary material for: Differential protein expression in diverse brain areas of Parkinson’s and Alzheimer’s disease patients
Source: Sci Rep. 2020 Aug 4;10:13149. doi: 10.1038/s41598-020-70174-z (PMC7403590; doi:10.1038/s41598-020-70174-z)
Supplement: Supplementary file 1 — Supplementary Information. [file 41598_2020_70174_MOESM1_ESM.pdf]

Differential protein expression in diverse brain areas of Parkinson's and Alzheimer's disease patients

AR Esteves[1,2] & SM Cardoso[1,2,3*]

[1]CNC–Center for Neuroscience and Cell Biology, [2]CIBB-Centre for Innovative Biomedicine and Biotechnology, [3]Institute of Cellular and Molecular Biology, Faculty of Medicine, University of Coimbra, Coimbra, Portugal

***Corresponding author:** CNC - Center for Neuroscience and Cell Biology, University of Coimbra, Largo Marquês de Pombal 3004-517 Coimbra, Portugal; Phone: +351 239 820190; Fax: +351-239-822776; E-mail: cardoso.sandra.m@gmail.com

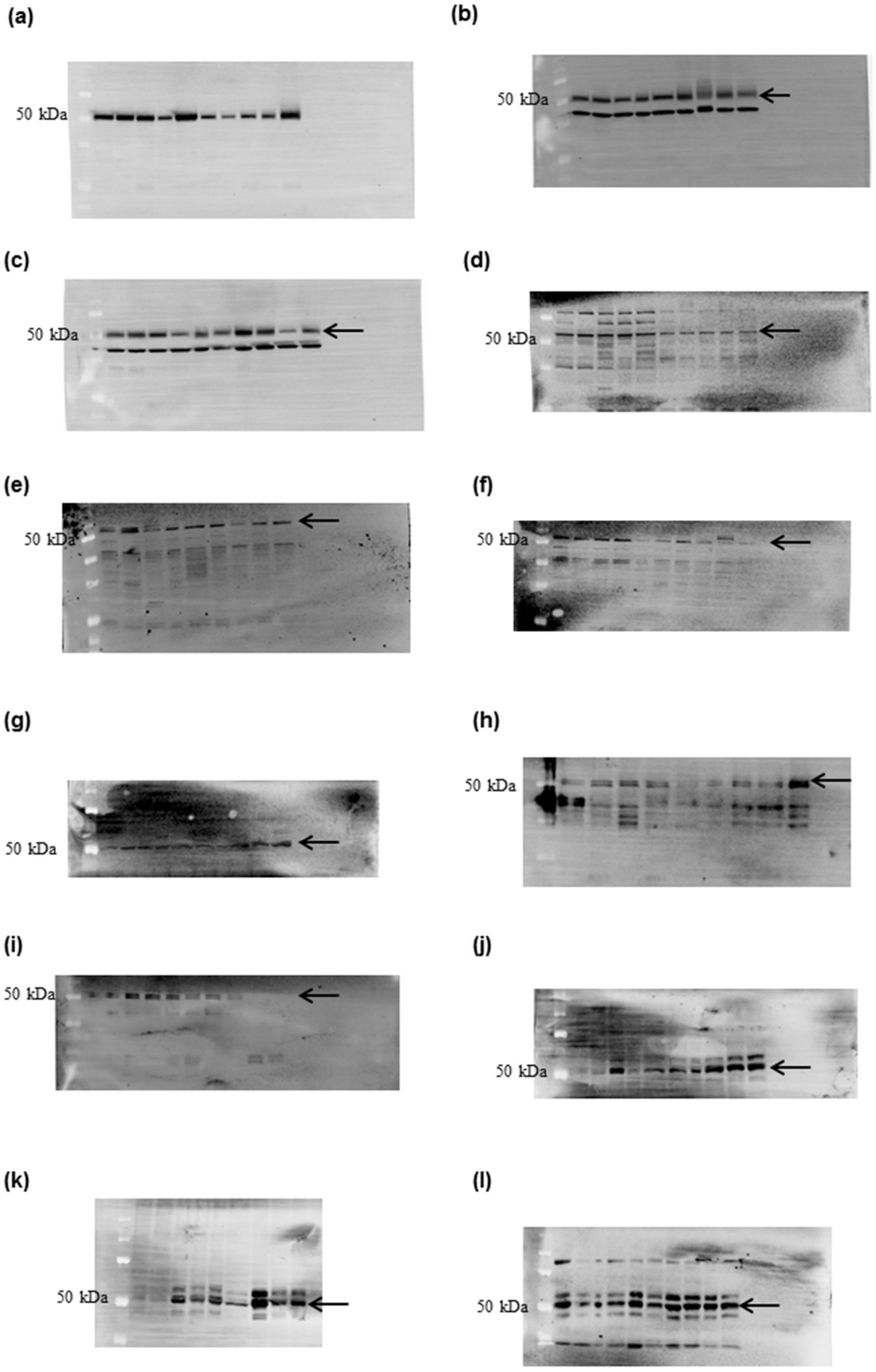
(a)
50 kDa
(b)
50 kDa
(c)
50 kDa
(d)
50 kDa
(e)
50 kDa
(f)
50 kDa
(g)
50 kDa
(h)
50 kDa
(i)
50 kDa
(j)
50 kDa
(k)
50 kDa
(l)
50 kDa

Supplementary Figure 1. (a) Whole representative western blot of PD SNpc acetylated α-tubulin; (b) Whole representative western blot of PD Hippocampus acetylated α-tubulin; (c) Whole representative western blot of PD Cortex acetylated α-tubulin; (d) Whole representative western blot of PD SNpc acetylated-tau; (e) Whole representative western blot of PD Hippocampus acetylated-tau; (f) Whole representative western blot of PD Cortex acetylated-tau; (g) Whole representative western blot of PD SNpc phospho-tau; (h) Whole representative western blot of PD Hippocampus phospho-tau; (i) Whole representative western blot of PD Cortex phospho-tau; (j) Whole representative western blot of PD SNpc Tau; (k) Whole representative western blot of PD Hippocampus Tau; (l) Whole representative western blot of PD Cortex Tau.

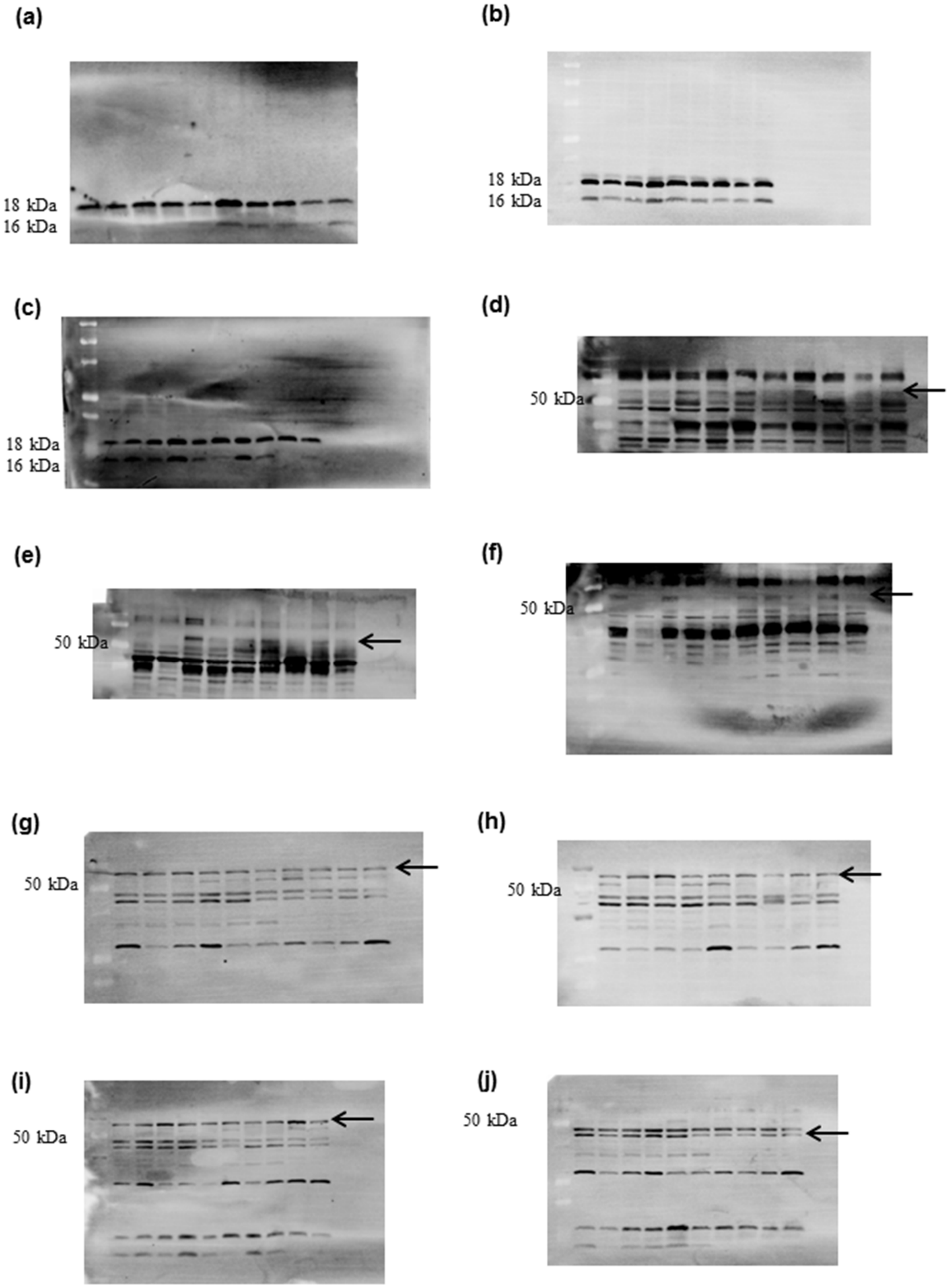
(a)
18 kDa
16 kDa
(b)
18 kDa
16 kDa
(c)
18 kDa
16 kDa
(d)
50 kDa
(e)
50 kDa
(f)
50 kDa
(g)
50 kDa
(h)
50 kDa
(i)
50 kDa
(j)
50 kDa

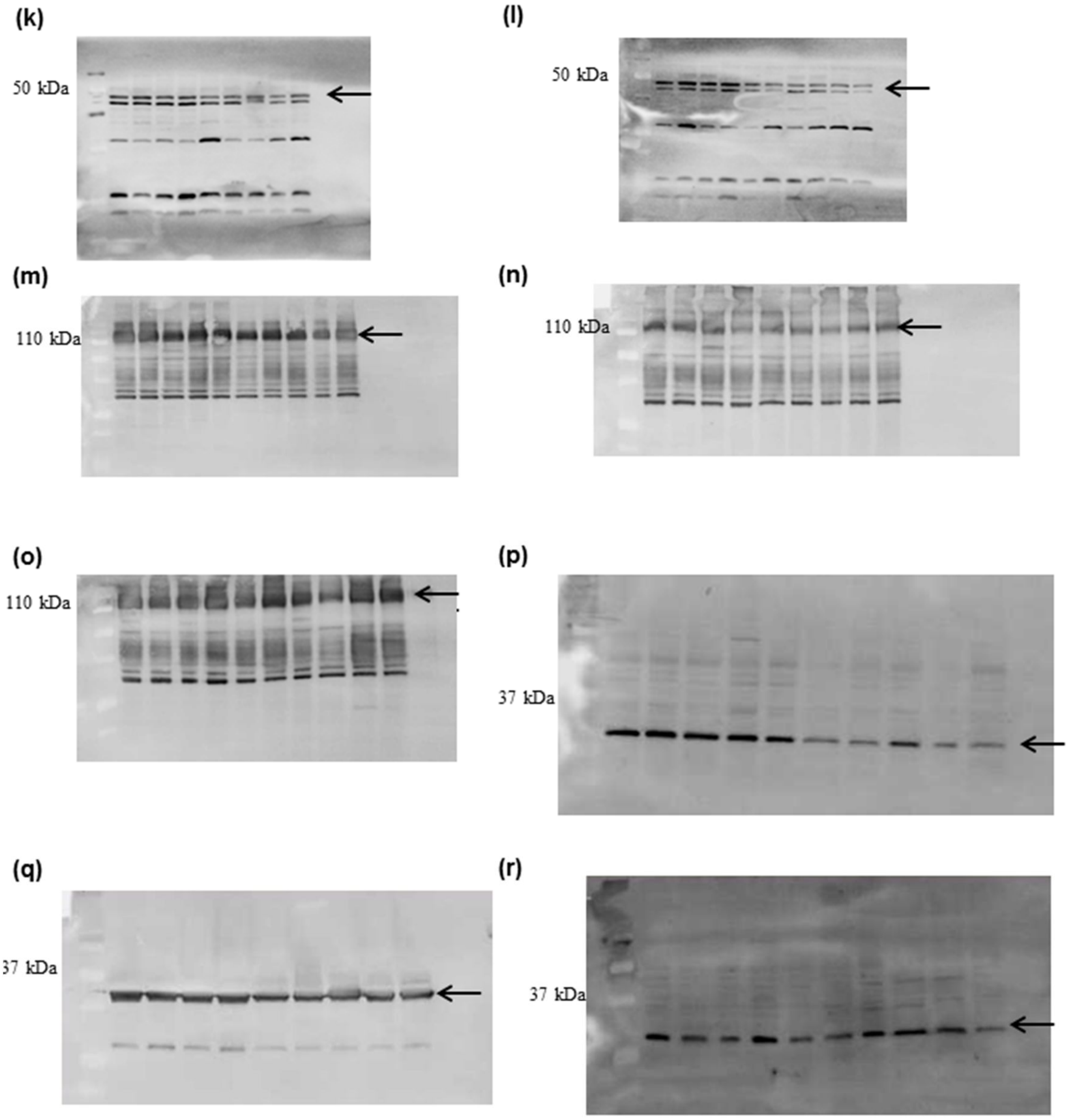

Supplementary Figure 2. (a) Whole representative western blot of PD SNpc LC3; (b) Whole representative western blot of PD Hippocampus LC3; (c) Whole representative western blot of PD Cortex LC3; (d) Whole representative western blot of PD SNpc p62; (e) Whole representative western blot of PD Hippocampus p62; (f) Whole representative western blot of PD Cortex p62; (g) Whole representative western blot of PD SNpc Beclin; (h) Whole representative western blot of PD Hippocampus Beclin; (i) Whole representative western blot of PD Cortex Beclin; (j) Whole representative western blot of PD SNpc Lamp2A; (k) Whole representative western blot of PD Hippocampus Lamp2A; (l) Whole representative western blot of PD Cortex Lamp2A; (m) Whole representative western blot of PD SNpc Lamp1; (n) Whole representative western blot of PD Hippocampus Lamp1; (o) Whole representative western blot of PD Cortex Lamp1; (p) Whole representative western blot of PD SNpc CathepsinD; (q) Whole representative western blot of PD Hippocampus CathepsinD; (r) Whole representative western blot of PD Cortex CathepsinD.

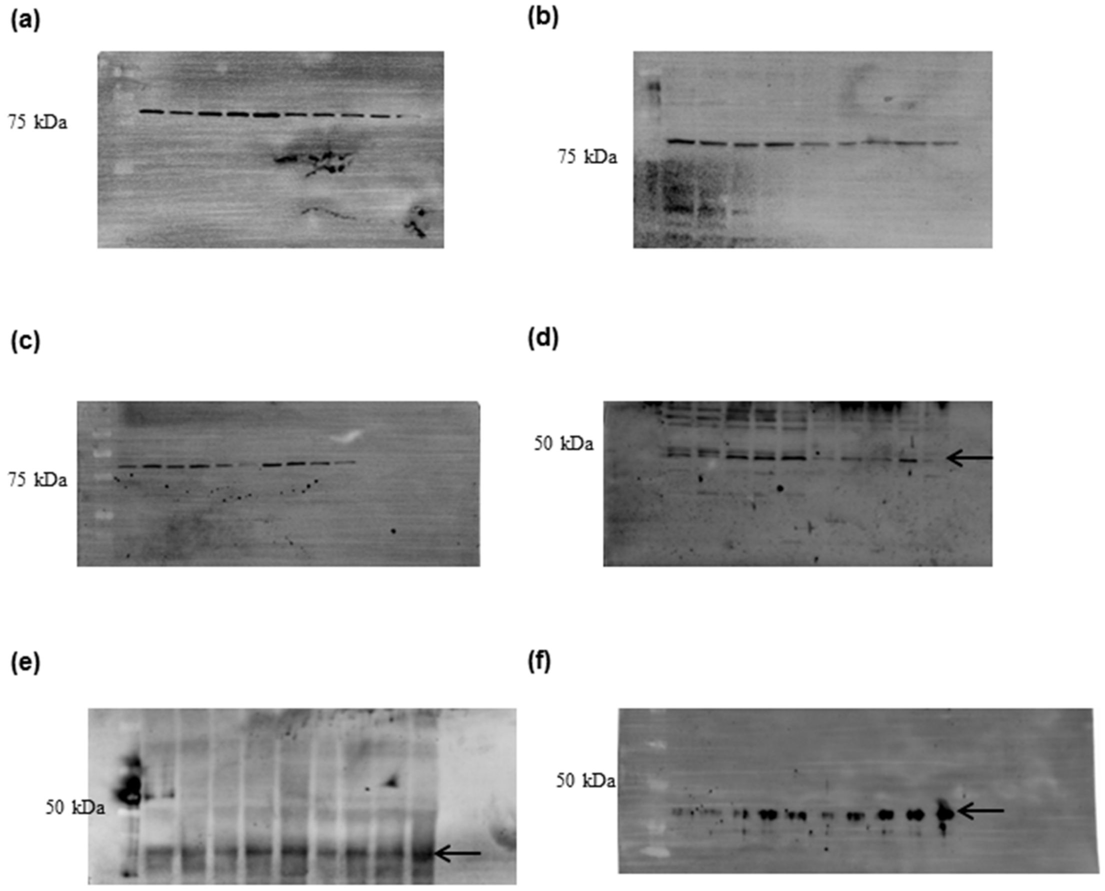

Supplementary Figure 3. (a) Whole representative western blot of PD SNpc GRP78; (b) Whole representative western blot of PD Hippocampus GRP78; (c) Whole representative western blot of PD Cortex GRP78; (d) Whole representative western blot of PD SNpc ATF4; (e) Whole representative western blot of PD Hippocampus ATF4; (f) Whole representative western blot of PD Cortex ATF4.

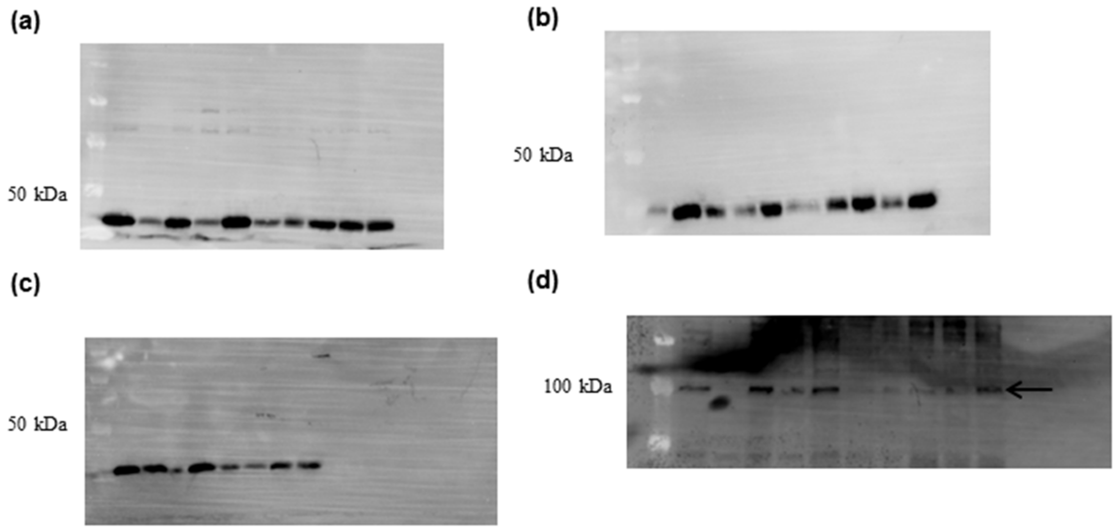

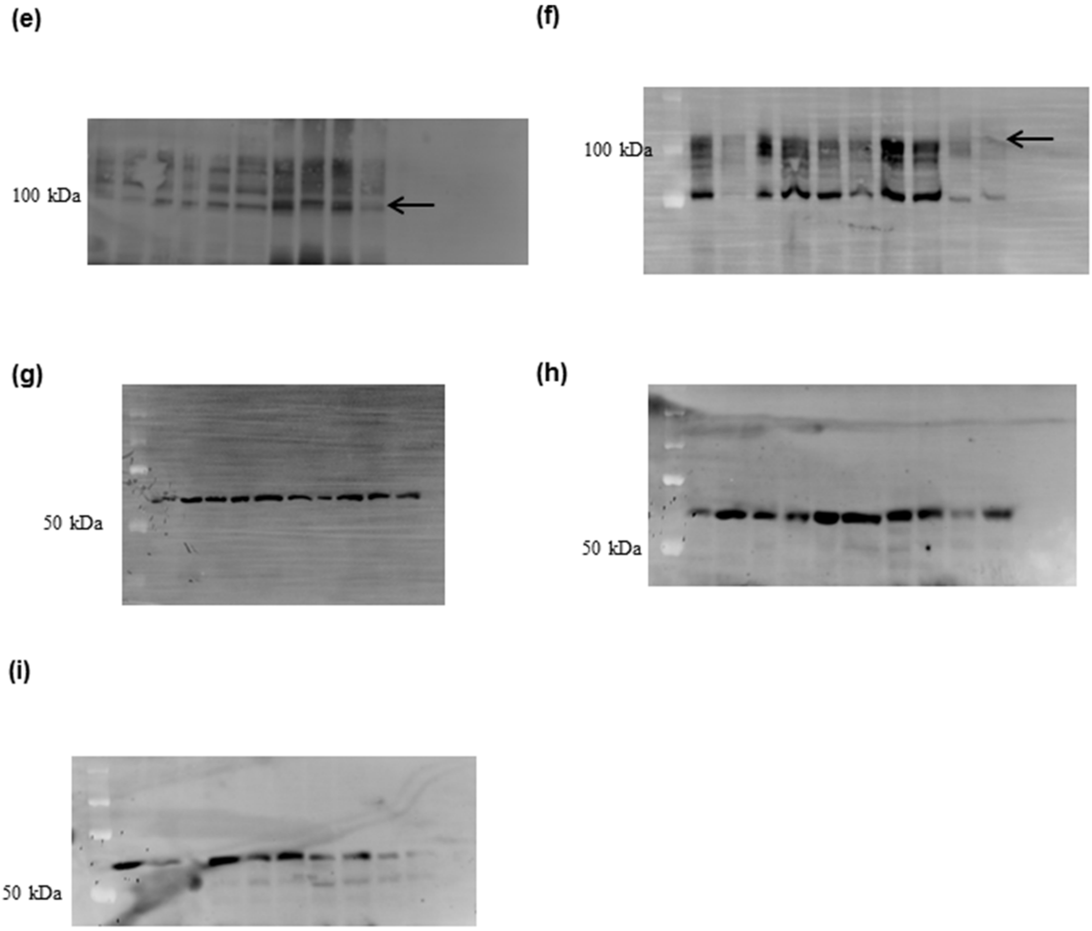

Supplementary Figure 4. (a) Whole representative western blot of PD SNpc Synaptophysin; (b) Whole representative western blot of PD Hippocampus Synaptophysin; (c) Whole representative western blot of PD Cortex Synaptophysin; (d) Whole representative western blot of PD SNpc PSD95; (e) Whole representative western blot of PD Hippocampus PSD95; (f) Whole representative western blot of PD Cortex PSD95; (g) Whole representative western blot of PD SNpc HSP60; (h) Whole representative western blot of PD Hippocampus HSP60; (i) Whole representative western blot of PD Cortex HSP60.

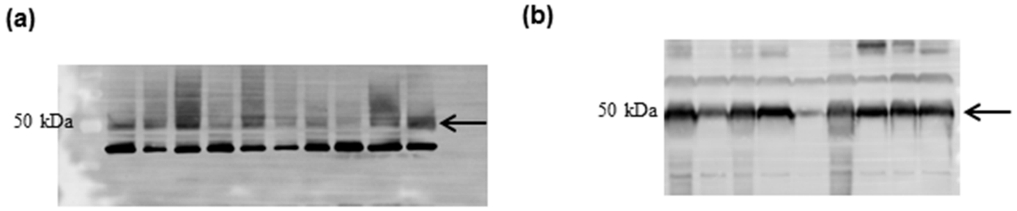

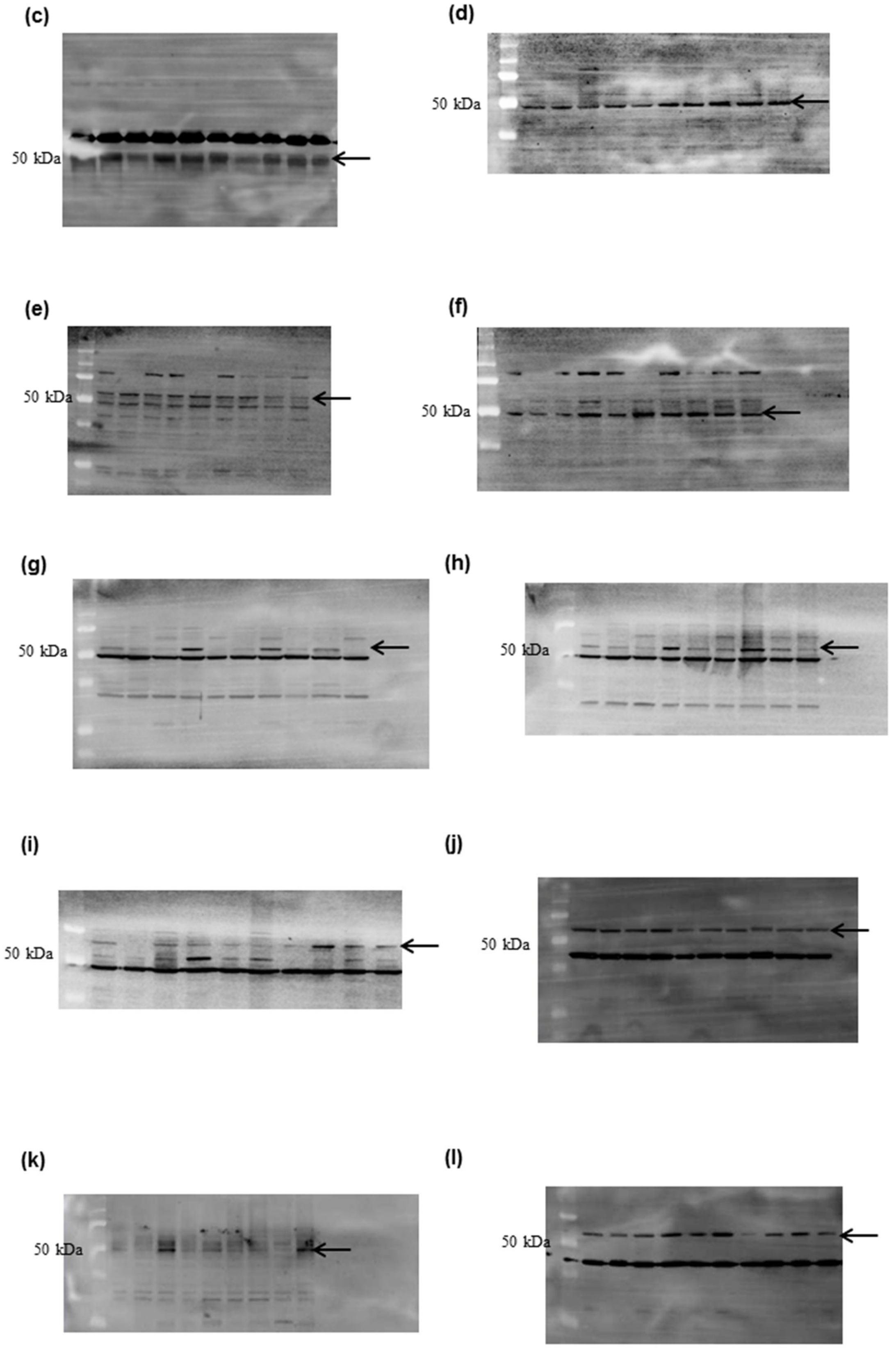

Supplementary Figure 5. (a) Whole representative western blot of AD SNpc acetylated α-tubulin; (b) Whole representative western blot of AD Hippocampus

acetylated α-tubulin; (c) Whole representative western blot of AD Cortex acetylated α-tubulin; (d) Whole representative western blot of AD SNpc acetylated-tau; (e) Whole representative western blot of AD Hippocampus acetylated-tau; (f) Whole representative western blot of AD Cortex acetylated-tau; (g) Whole representative western blot of AD SNpc phospho-tau; (h) Whole representative western blot of AD Hippocampus phospho-tau; (i) Whole representative western blot of AD Cortex phospho-tau; (j) Whole representative western blot of AD SNpc Tau; (k) Whole representative western blot of AD Hippocampus Tau; (l) Whole representative western blot of AD Cortex Tau.

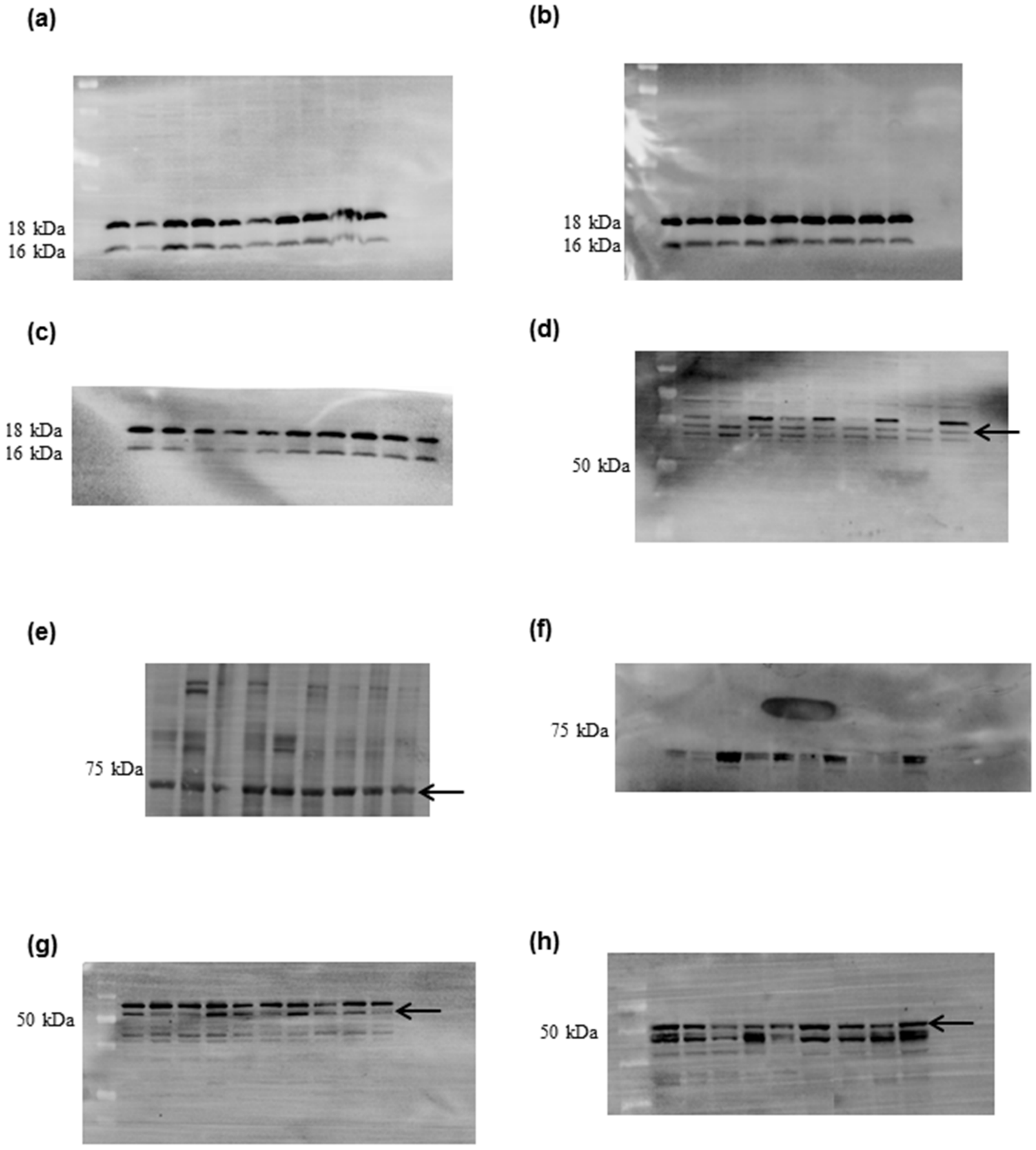

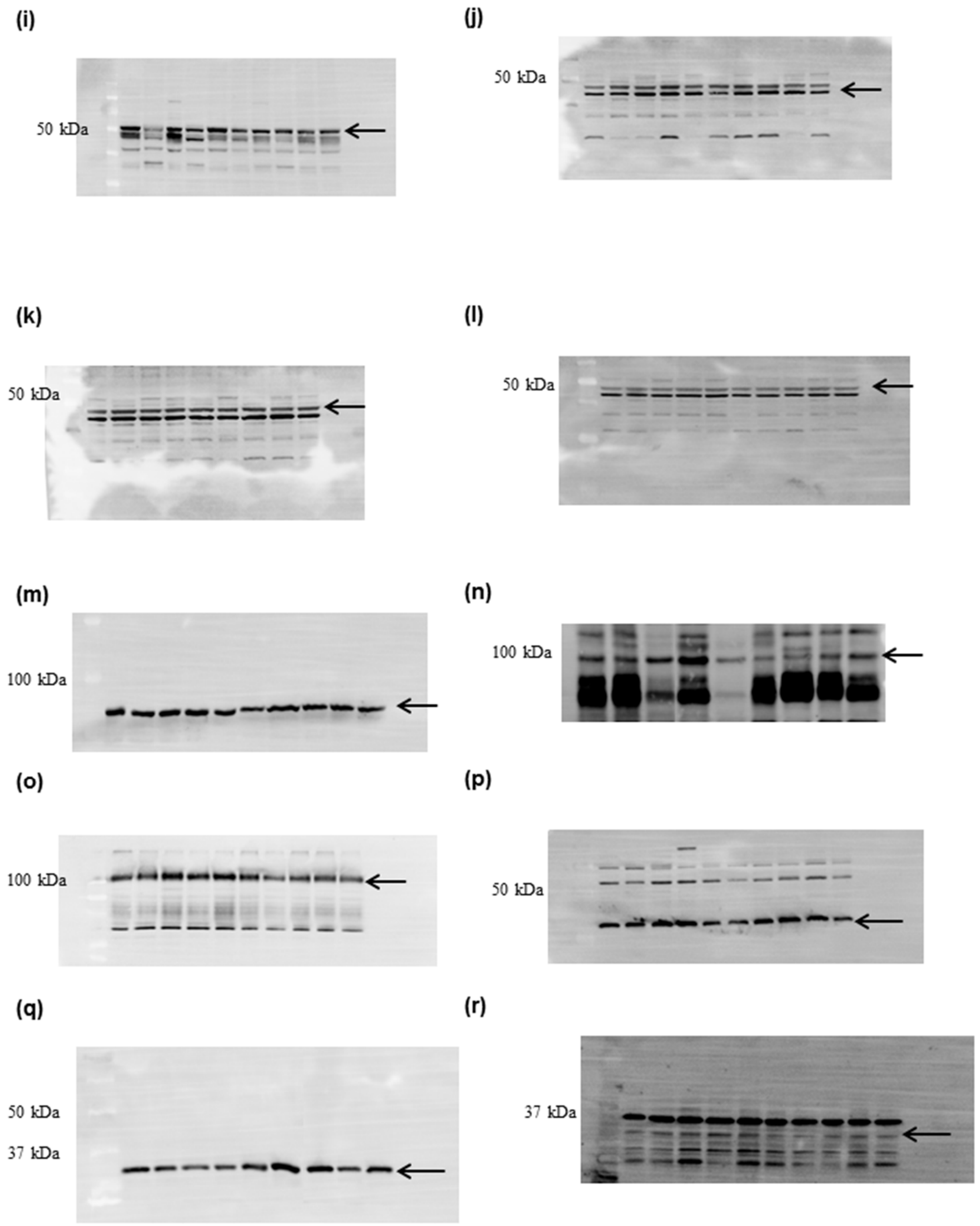

Supplementary Figure 6. (a) Whole representative western blot of AD SNpc LC3; (b) Whole representative western blot of AD Hippocampus LC3; (c) Whole representative western blot of AD Cortex LC3; (d) Whole representative western blot of AD SNpc p62; (e) Whole representative western blot of AD Hippocampus p62; (f) Whole representative western blot of AD Cortex p62; (g) Whole representative western blot of AD SNpc Beclin; (h) Whole representative western blot of AD Hippocampus Beclin; (i)

Whole representative western blot of AD Cortex Beclin; (j) Whole representative western blot of AD SNpc Lamp2A; (k) Whole representative western blot of AD Hippocampus Lamp2A; (l) Whole representative western blot of AD Cortex Lamp2A; (m) Whole representative western blot of AD SNpc Lamp1; (n) Whole representative western blot of AD Hippocampus Lamp1; (o) Whole representative western blot of AD Cortex Lamp1; (p) Whole representative western blot of AD SNpc CathepsinD; (q) Whole representative western blot of AD Hippocampus CathepsinD; (r) Whole representative western blot of AD Cortex CathepsinD.

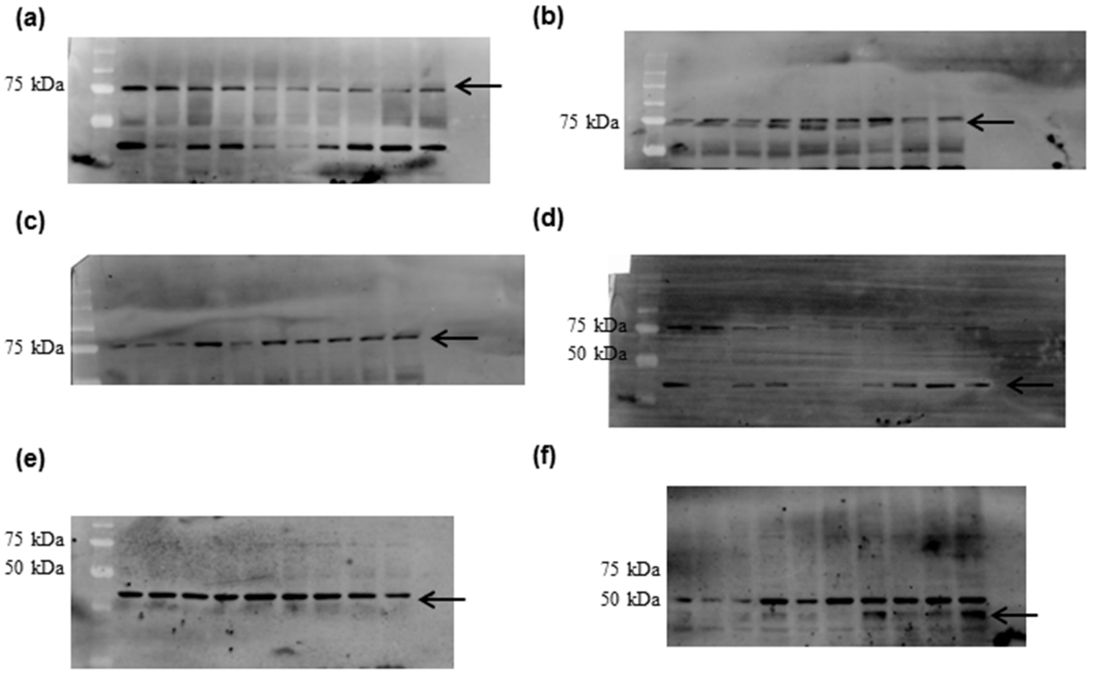

Supplementary Figure 7. (a) Whole representative western blot of AD SNpc GRP78; (b) Whole representative western blot of AD Hippocampus GRP78; (c) Whole representative western blot of AD Cortex GRP78; (d) Whole representative western blot of AD SNpc ATF4; (e) Whole representative western blot of AD Hippocampus ATF4; (f) Whole representative western blot of AD Cortex ATF4.

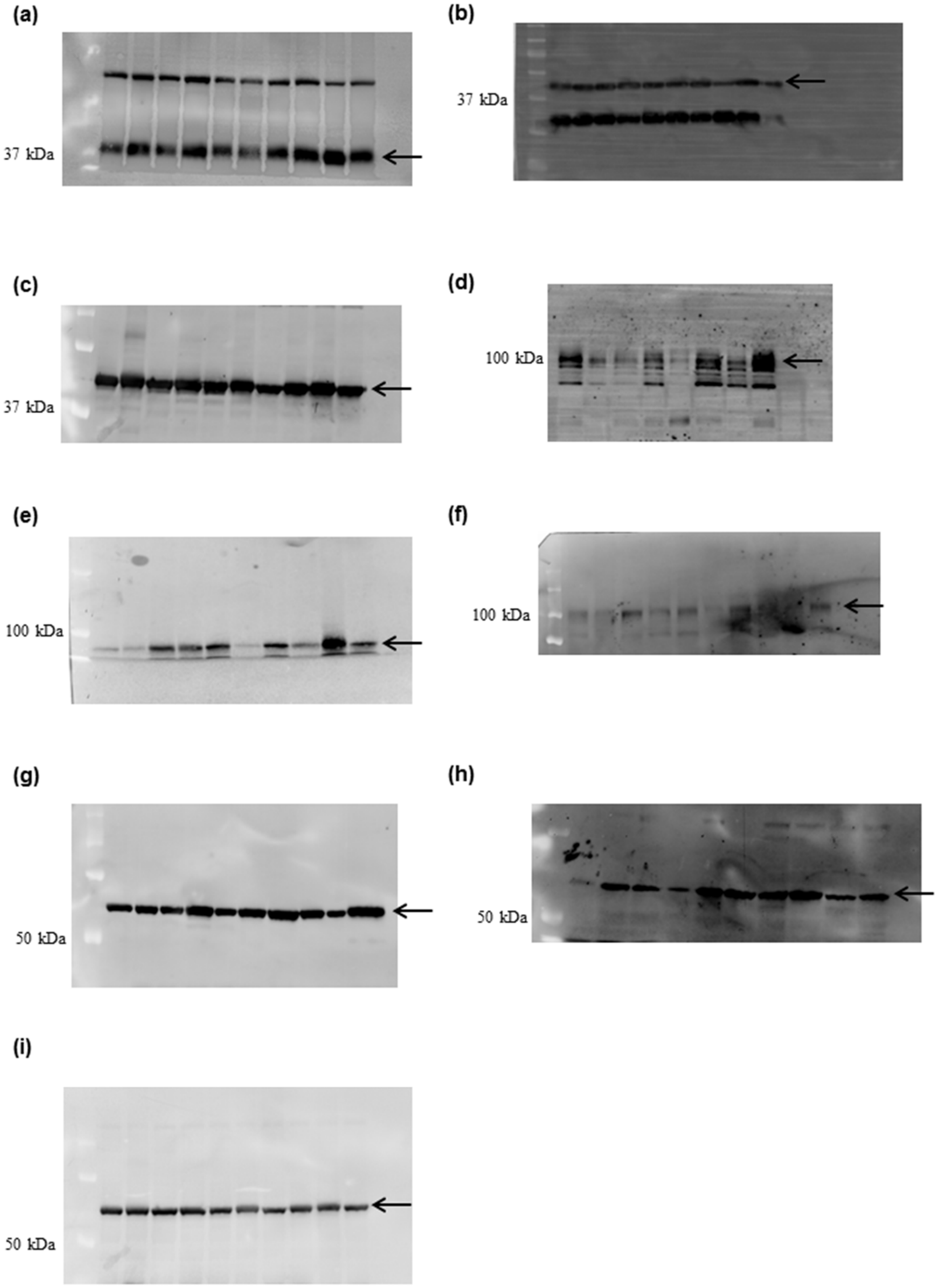

Supplementary Figure 8. (a) Whole representative western blot of AD SNpc Synaptophysin; (b) Whole representative western blot of AD Hippocampus Synaptophysin; (c) Whole representative western blot of AD Cortex Synaptophysin; (d) Whole representative western blot of AD SNpc PSD95; (e) Whole representative

western blot of AD Hippocampus PSD95; (f) Whole representative western blot of AD Cortex PSD95; (g) Whole representative western blot of AD SNpc HSP60; (h) Whole representative western blot of AD Hippocampus HSP60; (i) Whole representative western blot of AD Cortex HSP60.

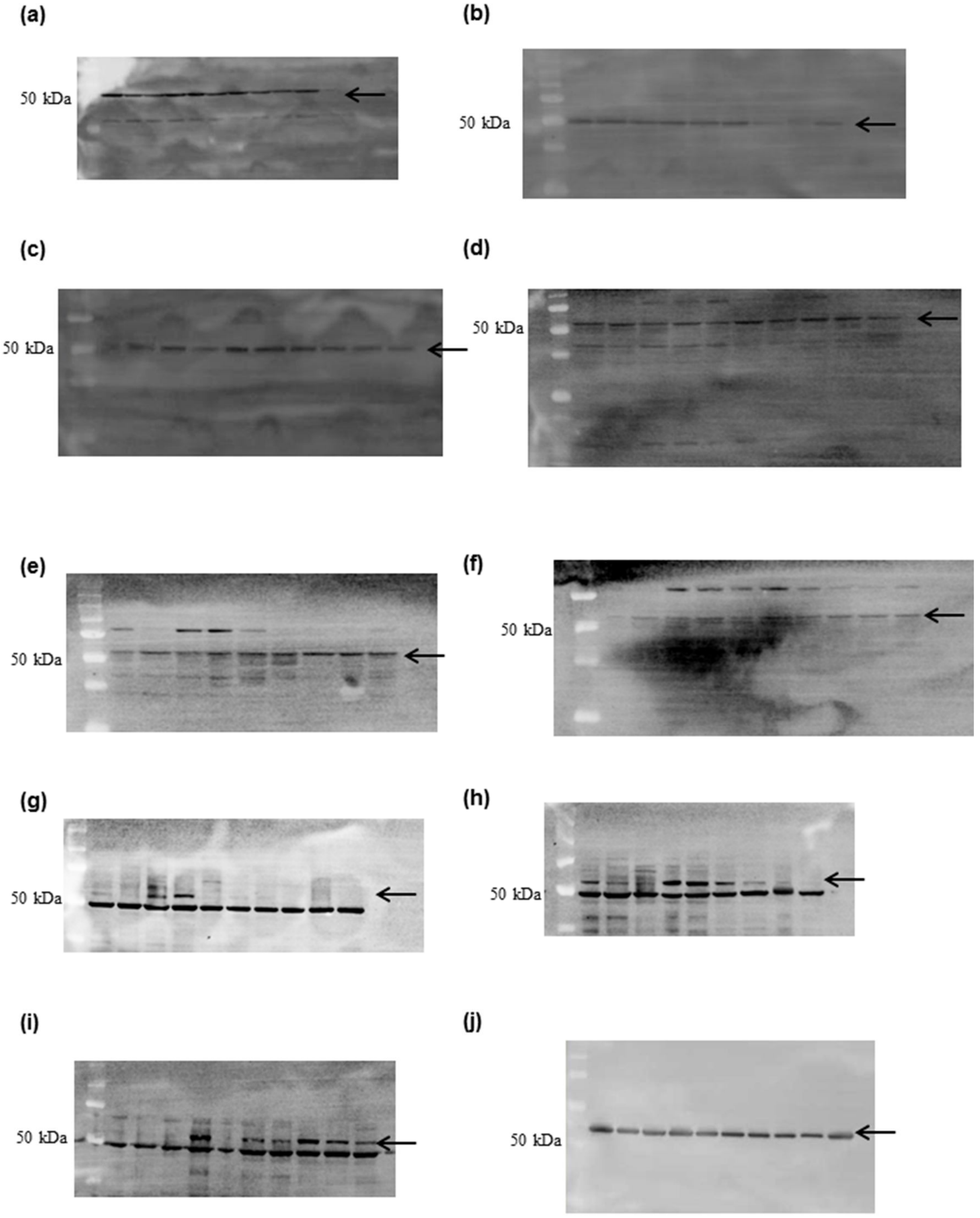

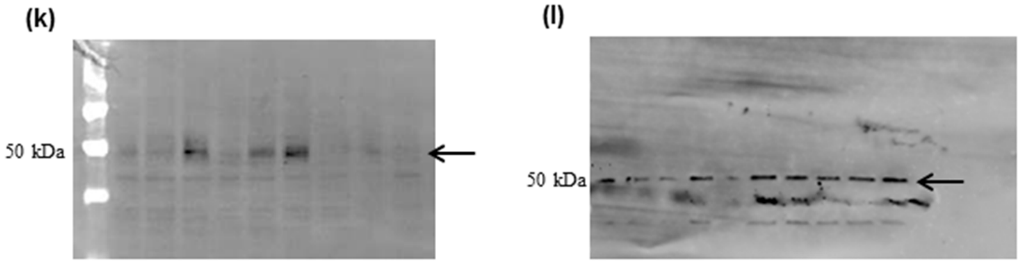

Supplementary Figure 9. (a) Whole representative western blot of DV SNpc acetylated α-tubulin; (b) Whole representative western blot of DV Hippocampus acetylated α-tubulin; (c) Whole representative western blot of DV Cortex acetylated α-tubulin; (d) Whole representative western blot of DV SNpc acetylated-tau; (e) Whole representative western blot of DV Hippocampus acetylated-tau; (f) Whole representative western blot of DV Cortex acetylated-tau; (g) Whole representative western blot of DV SNpc phospho-tau; (h) Whole representative western blot of DV Hippocampus phospho-tau; (i) Whole representative western blot of DV Cortex phospho-tau; (j) Whole representative western blot of DV SNpc Tau; (k) Whole representative western blot of DV Hippocampus Tau; (l) Whole representative western blot of DV Cortex Tau.

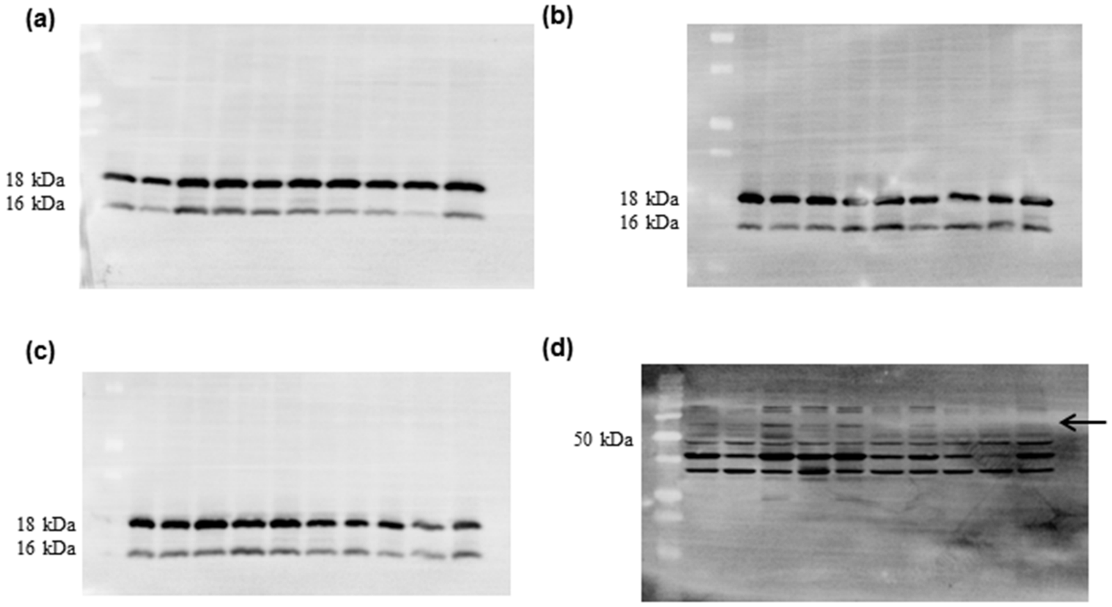

(e)

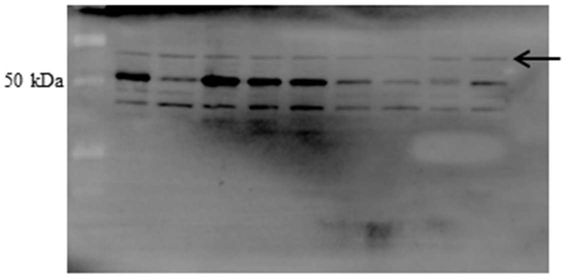
50 kDa

(f)

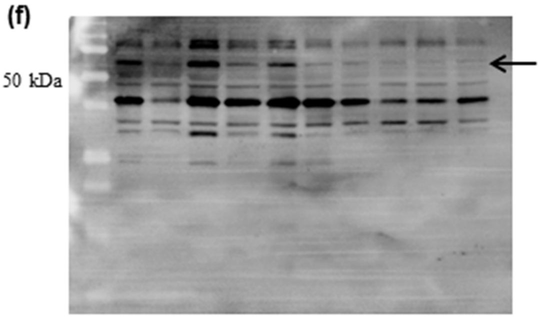
50 kDa

(g)

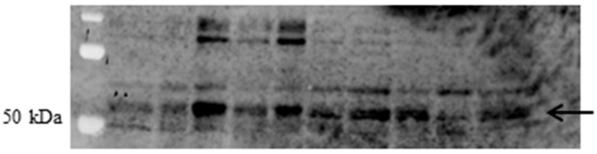
50 kDa

(h)

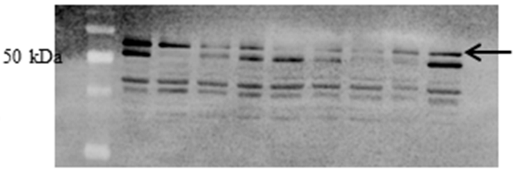
50 kDa

(i)

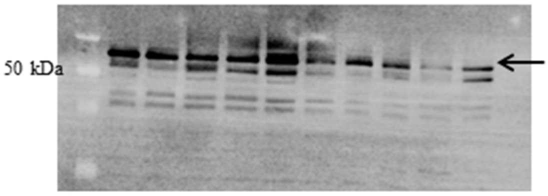
50 kDa

(j)

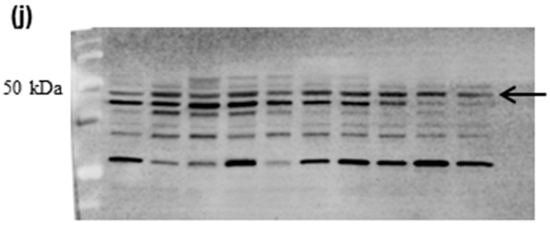
50 kDa

(k)

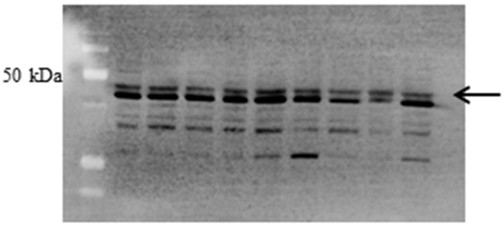
50 kDa

(l)

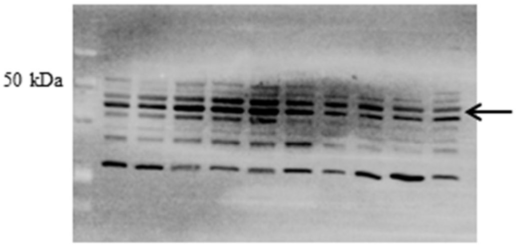
50 kDa

(m)

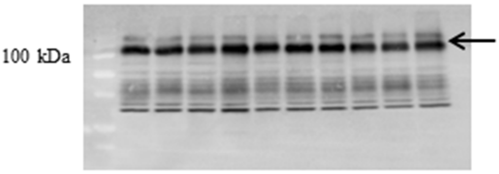
100 kDa

(n)

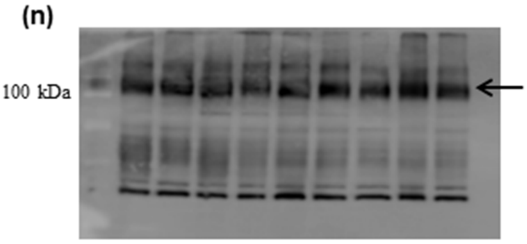
100 kDa

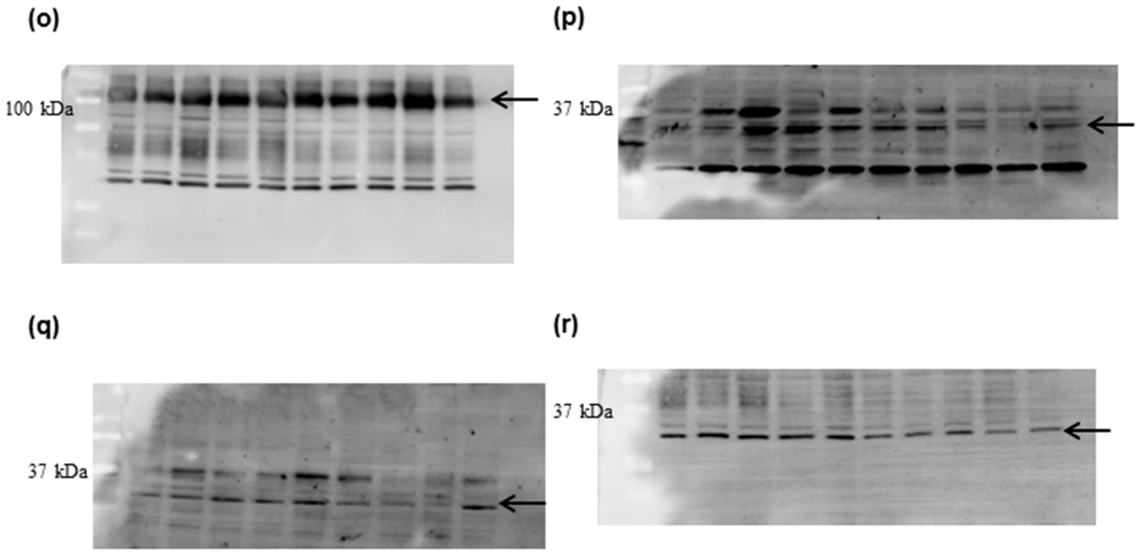

Supplementary Figure 10. (a) Whole representative western blot of DV SNpc LC3; (b) Whole representative western blot of DV Hippocampus LC3; (c) Whole representative western blot of DV Cortex LC3; (d) Whole representative western blot of DV SNpc p62; (e) Whole representative western blot of DV Hippocampus p62; (f) Whole representative western blot of DV Cortex p62; (g) Whole representative western blot of DV SNpc Beclin; (h) Whole representative western blot of DV Hippocampus Beclin; (i) Whole representative western blot of DV Cortex Beclin; (j) Whole representative western blot of DV SNpc Lamp2A; (k) Whole representative western blot of DV Hippocampus Lamp2A; (l) Whole representative western blot of DV Cortex Lamp2A; (m) Whole representative western blot of DV SNpc Lamp1; (n) Whole representative western blot of DV Hippocampus Lamp1; (o) Whole representative western blot of DV Cortex Lamp1; (p) Whole representative western blot of DV SNpc CathepsinD; (q) Whole representative western blot of DV Hippocampus CathepsinD; (r) Whole representative western blot of DV Cortex CathepsinD.

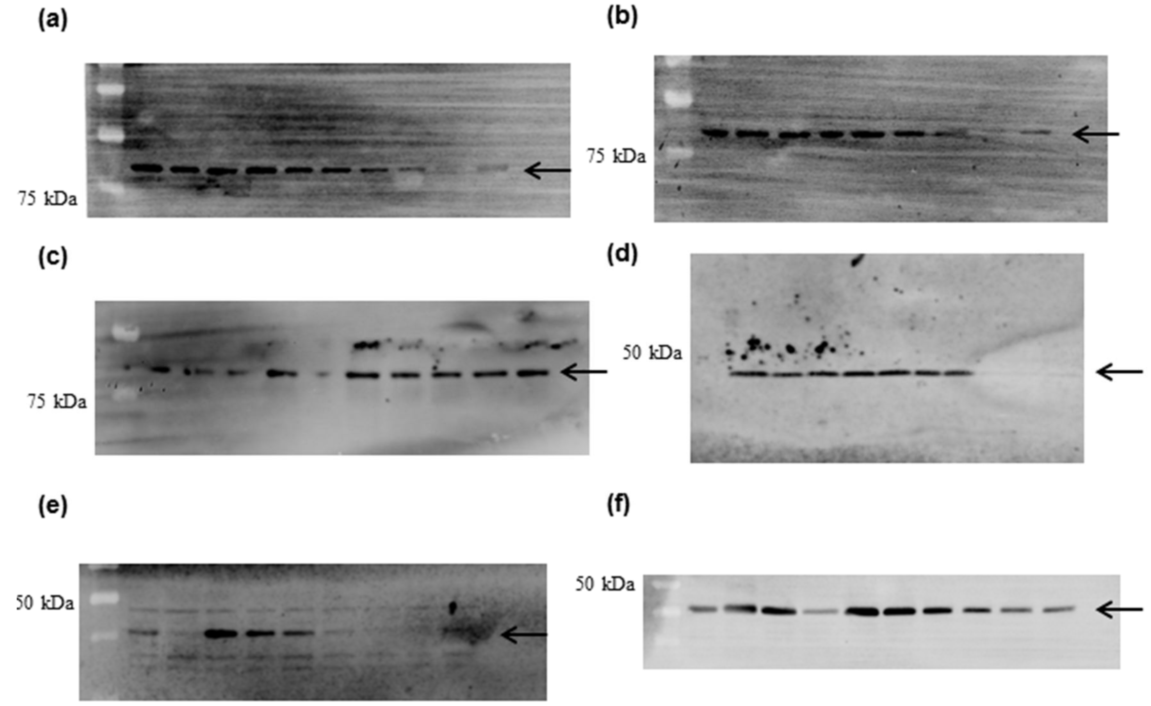

Supplementary Figure 11. (a) Whole representative western blot of DV SNpc GRP78; (b) Whole representative western blot of DV Hippocampus GRP78; (c) Whole representative western blot of DV Cortex GRP78; (d) Whole representative western blot of DV SNpc ATF4; (e) Whole representative western blot of DV Hippocampus ATF4; (f) Whole representative western blot of DV Cortex ATF4.

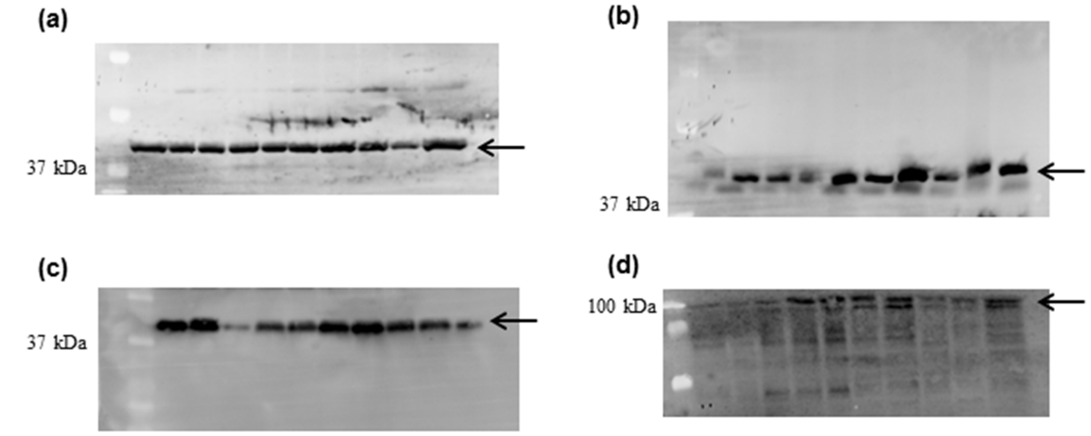

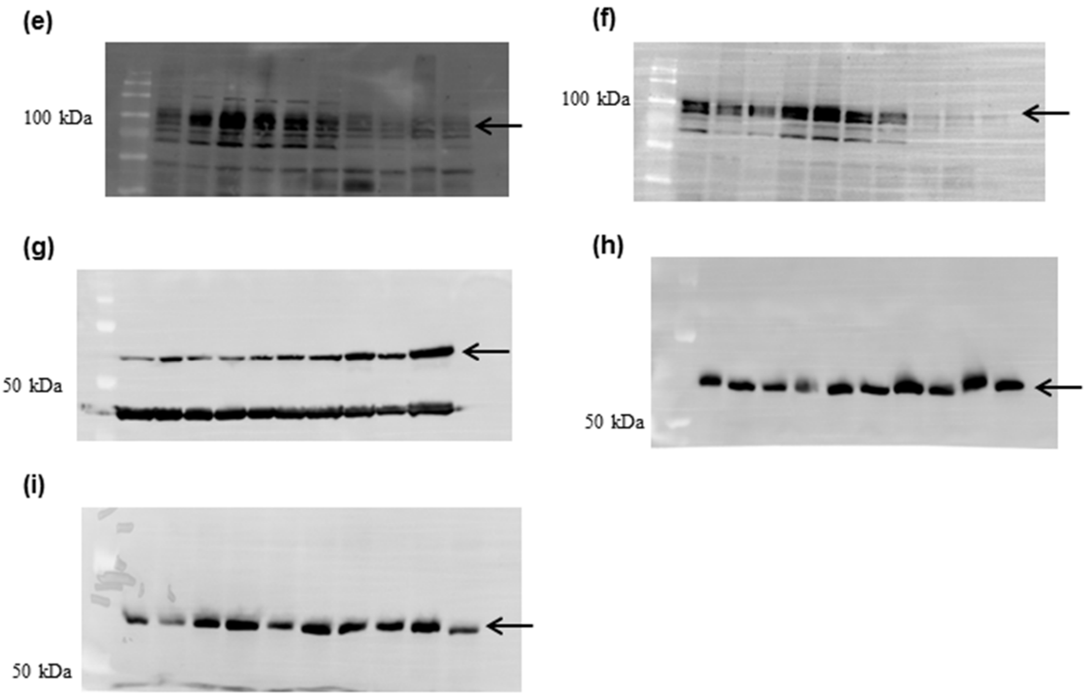

Supplementary Figure 12. (a) Whole representative western blot of DV SNpc Synaptophysin; (b) Whole representative western blot of DV Hippocampus Synaptophysin; (c) Whole representative western blot of DV Cortex Synaptophysin; (d) Whole representative western blot of DV SNpc PSD95; (e) Whole representative western blot of DV Hippocampus PSD95; (f) Whole representative western blot of DV Cortex PSD95; (g) Whole representative western blot of DV SNpc; (h) HSP60Whole representative western blot of DV Hippocampus HSP60; (i) Whole representative western blot of DV Cortex HSP60.

Before probing membranes with the loading control protein (alpha-tubulin) stripping was performed for the removal of primary and secondary antibodies from the western blot membrane. Stripping protocol was the following: -incubation for 30min with Methanol 40% to remove the excess of ECF; -incubation with Tris buffered saline solution supplemented with Tween (TBS-T) for 30min; -incubation for 5min with water, followed by 10min with 0,2M NaOH and 5min with water to remove the primary and secondary antibody; finally membranes were blocked with 5% BSA in TBS-T and incubated with the loading control antibody overnight. Full length blots of alpha-tubulin band are showed in the next Supplementary Figures. For Figure 13, 14 and 15 from (g) to (l) membranes were cut to separately blot alpha-tubulin to avoid Aβ and SNCA background staining allowing a clearer quantification.

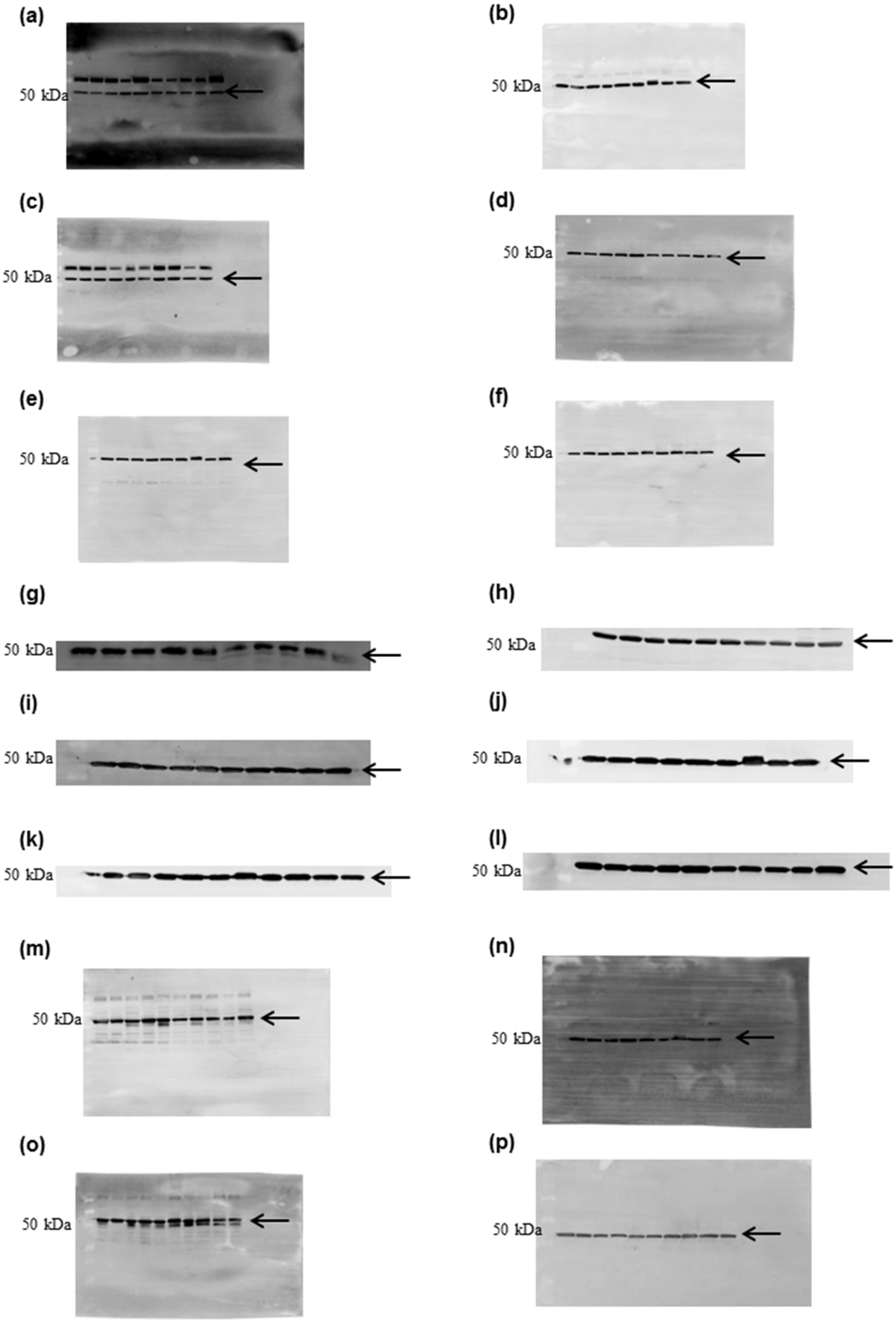

Supplementary Figure 13. (a) Whole representative western blot of PD SNpc alpha-tubulin for Figure 1(a). (b) Whole representative western blot of PD Hippocampus alpha-tubulin for Figure 1(b). (c) Whole representative western blot of PD Cortex alpha-

tubulin for Figure 1(c). (d) Whole representative western blot of PD SNpc alpha-tubulin for Figure 2(a). (e) Whole representative western blot of PD Hippocampus alpha-tubulin for Figure 2(b). (f) Whole representative western blot of PD Cortex alpha-tubulin for Figure 2(c). (g) Whole representative western blot of PD SNpc alpha-tubulin for Figure 3(a). (h) Whole representative western blot of PD Hippocampus alpha-tubulin for Figure 3(b). (i) Whole representative western blot of PD Cortex alpha-tubulin for Figure 3(c). (j) Whole representative western blot of PD SNpc alpha-tubulin for Figure 3(e). (k) Whole representative western blot of PD Hippocampus alpha-tubulin for Figure 3(f). (l) Whole representative western blot of PD Cortex alpha-tubulin for Figure 3(g). (m) Whole representative western blot of PD SNpc alpha-tubulin for Figure 4(a) and Figure 5(a). (n) Whole representative western blot of PD Hippocampus alpha-tubulin for Figure 4(b). (o) Whole representative western blot of PD Cortex alpha-tubulin for Figure 4(c) and Figure 5(c). (p) Whole representative western blot of PD Hippocampus alpha-tubulin for Figure 5(b).

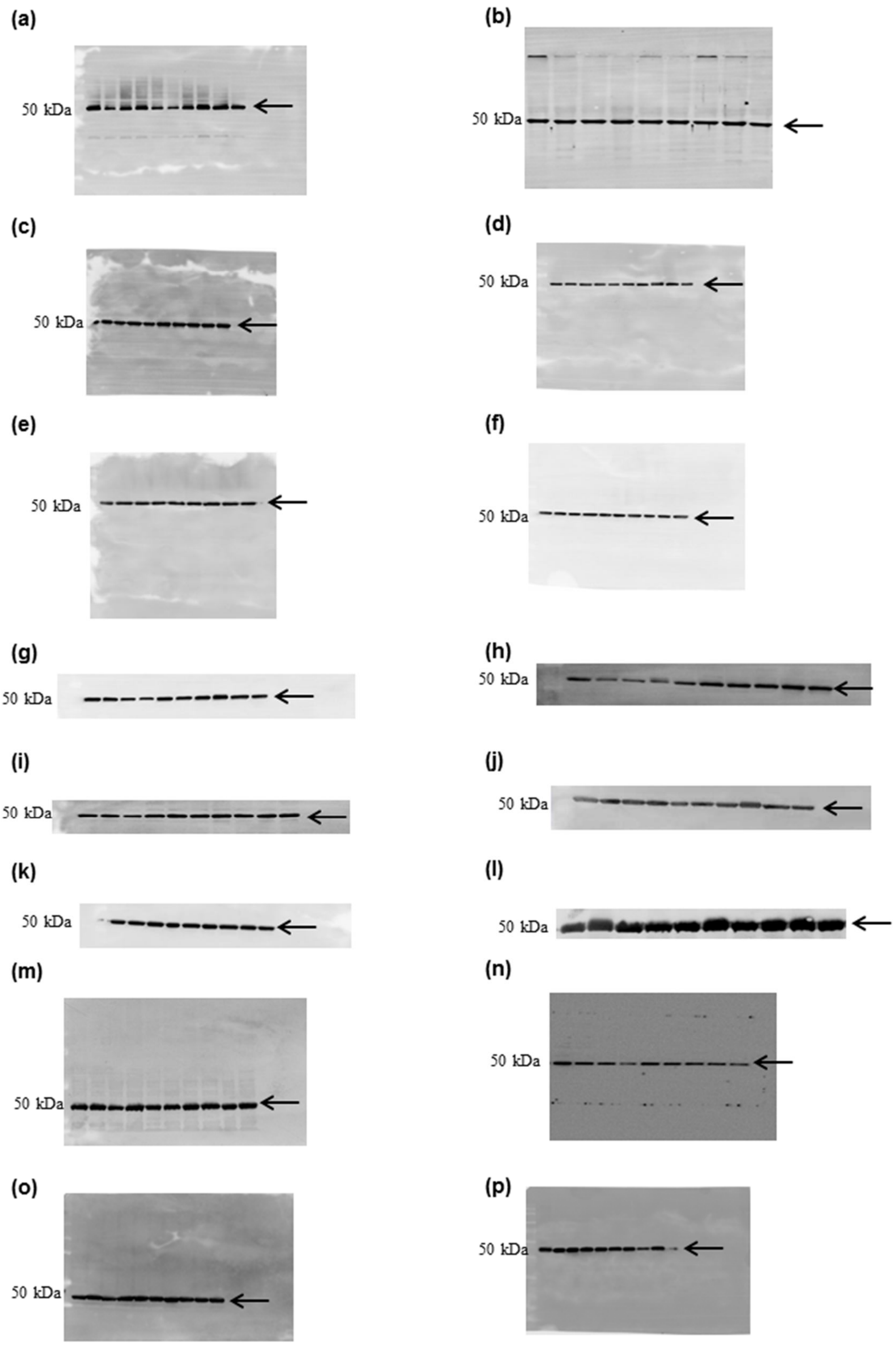

Supplementary Figure 14. (a) Whole representative western blot of AD SNpc alpha-tubulin for Figure 6(a). (b) Whole representative western blot of AD Hippocampus alpha-tubulin for Figure 6(b). (c) Whole representative western blot of AD Cortex

alpha-tubulin for Figure 6(c). (d) Whole representative western blot of AD SNpc alpha-tubulin for Figure 7(a). (e) Whole representative western blot of AD Hippocampus alpha-tubulin for Figure 7(b). (f) Whole representative western blot of AD Cortex alpha-tubulin for Figure 7(c). (g) Whole representative western blot of AD SNpc alpha-tubulin for Figure 8(a). (h) Whole representative western blot of AD Hippocampus alpha-tubulin for Figure 8(b). (i) Whole representative western blot of AD Cortex alpha-tubulin for Figure 8(c). (j) Whole representative western blot of AD SNpc alpha-tubulin for Figure 8(e). (k) Whole representative western blot of AD Hippocampus alpha-tubulin for Figure 8(f). (l) Whole representative western blot of AD Cortex alpha-tubulin for Figure 8(g). (m) Whole representative western blot of AD SNpc alpha-tubulin for Figure 9(a) and Figure 10(a). (n) Whole representative western blot of AD Hippocampus alpha-tubulin for Figure 9(b). (o) Whole representative western blot of AD Cortex alpha-tubulin for Figure 9(c) and Figure 10(c). (p) Whole representative western blot of AD Hippocampus alpha-tubulin for Figure 10(b).

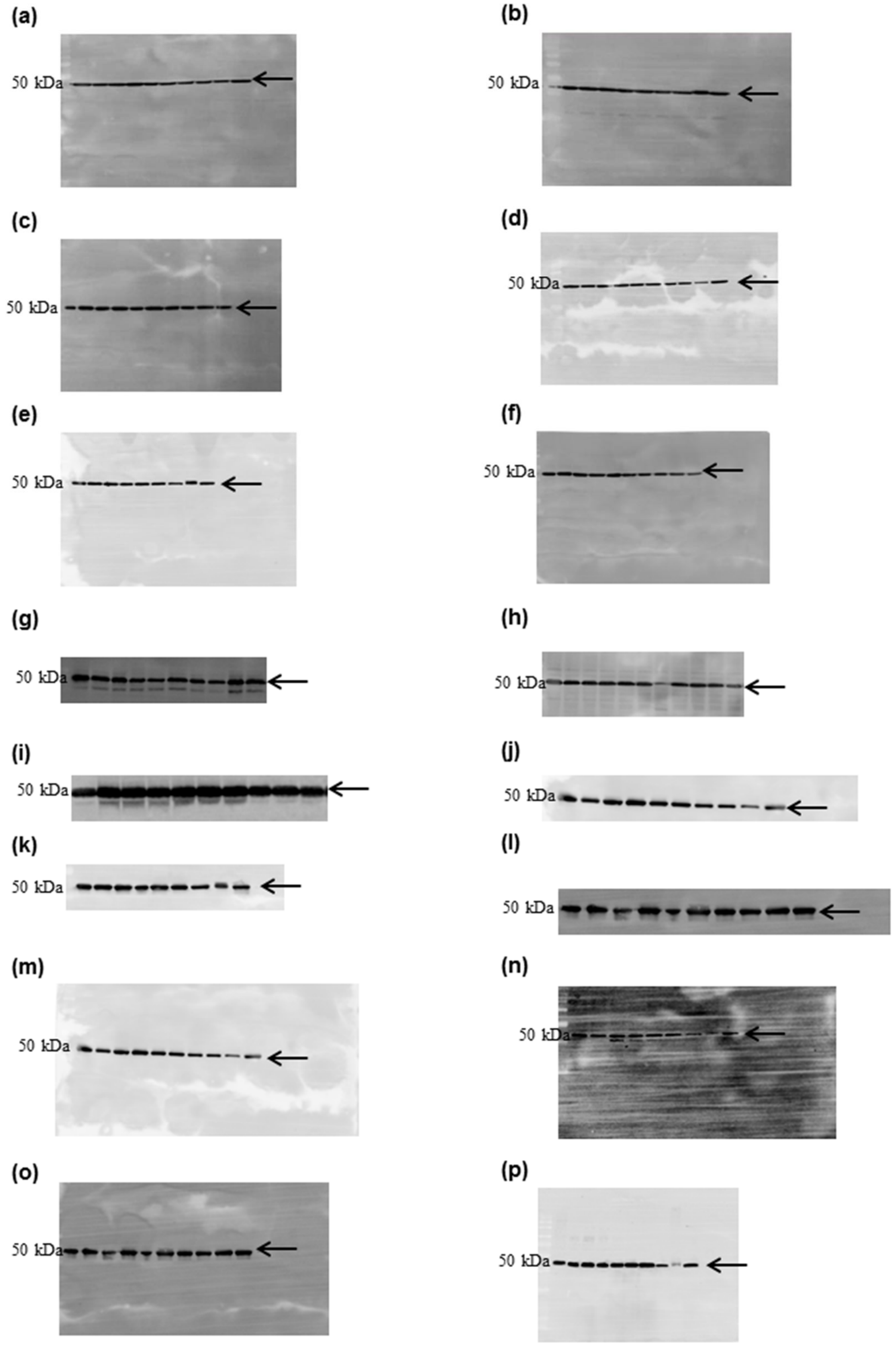

Supplementary Figure 15. (a) Whole representative western blot of VD SNpc alpha-tubulin for Figure 11(a). (b) Whole representative western blot of VD Hippocampus alpha-tubulin for Figure 11(b). (c) Whole representative western blot of VD Cortex alpha-tubulin for Figure 11(c). (d) Whole representative western blot of VD SNpc alpha-tubulin for Figure 12(a). (e) Whole representative western blot of VD

Hippocampus alpha-tubulin for Figure 12(b). (f) Whole representative western blot of VD Cortex alpha-tubulin for Figure 12(c). (g) Whole representative western blot of VD SNpc alpha-tubulin for Figure 13(a). (h) Whole representative western blot of VD Hippocampus alpha-tubulin for Figure 13(b). (i) Whole representative western blot of VD Cortex alpha-tubulin for Figure 13(c). (j) Whole representative western blot of VD SNpc alpha-tubulin for Figure 13(e). (k) Whole representative western blot of VD Hippocampus alpha-tubulin for Figure 13(f). (l) Whole representative western blot of VD Cortex alpha-tubulin for Figure 13(g). (m) Whole representative western blot of VD SNpc alpha-tubulin for Figure 14(a) and Figure 15(a). (n) Whole representative western blot of VD Hippocampus alpha-tubulin for Figure 14(b). (o) Whole representative western blot of VD Cortex alpha-tubulin for Figure 14(c) and Figure 15(c). (p) Whole representative western blot of VD Hippocampus alpha-tubulin for Figure 15(b).
